# Transcriptomic profiling of *Solanum peruvianum* LA3858 revealed a *Mi-3*-mediated hypersensitive response to *Meloidogyne incognita*

**DOI:** 10.1186/s12864-020-6654-5

**Published:** 2020-03-23

**Authors:** Chong Du, Jingbin Jiang, He Zhang, Tingting Zhao, Huanhuan Yang, Dongye Zhang, Zhentong Zhao, Xiangyang Xu, Jingfu Li

**Affiliations:** Laboratory of Genetic Breeding in Tomato, College of Horticulture and Landscape Architecture, Northeast Agricultural University, Harbin, 150030 People’s Republic of China

**Keywords:** RNA sequencing, Root-knot nematode, *Mi-3*, ROS, Soil temperature

## Abstract

**Background:**

The *Mi-1* gene was the first identified and cloned gene that provides resistance to root-knot nematodes (RKNs) in cultivated tomato. However, owing to its temperature sensitivity, this gene does not meet the need for breeding disease-resistant plants that grow under high temperature. In this study, *Mi-3* was isolated from the wild species PI 126443 (LA3858) and was shown to display heat-stable resistance to RKNs. However, the mechanism that regulates this resistance remains unknown.

**Results:**

In this study, 4760, 1024 and 137 differentially expressed genes (DEGs) were enriched on the basis of pairwise comparisons (34 °C vs. 25 °C) at 0 (before inoculation), 3 and 6 days post-inoculation (dpi), respectively. A total of 7035 DEGs were identified from line LA3858 in the respective groups under the different soil temperature treatments. At 3 dpi, most DEGs were enriched in Kyoto Encyclopedia of Genes and Genomes (KEGG) pathways related to plant biotic responses, such as “plant-pathogen interaction” and “plant hormone signal transduction”. Significantly enriched DEGs were found to encode key proteins such as R proteins and heat-shock proteins (HSPs). Moreover, other DEGs were found to participate in Ca^2+^ signal transduction; the production of ROS; DEGs encoding transcription factors (TFs) from the bHLH, TGA, ERF, heat-shock transcription factor (HSF) and WRKY families were highly expressed, which contribute to be involved into the formation of phytohormones, such as salicylic acid (SA), jasmonic acid (JA) and ethylene (ET), the expression of most was upregulated at 3 dpi at the 25 °C soil temperature compared with the 34 °C soil temperature.

**Conclusion:**

Taken together, the results of our study revealed reliable candidate genes from wild materials LA3858, that are related to *Mi-3-*mediate resistance to *Meloidogyne incognita*. A large number of vital pathways and DEGs were expressed specifically in accession LA3858 grown at 34 °C and 25 °C soil temperatures at 3 dpi. Upon infection by RKNs, pattern-recognition receptors (PRRs) specifically recognized conserved pathogen-associated molecular patterns (PAMPs) as a result of pathogen-triggered immunity (PTI), and the downstream defensive signal transduction pathway was likely activated through Ca^2+^ signal channels. The expression of various TFs was induced to synthesize phytohormones and activate R proteins related to resistance, resulting in the development of effector-triggered immunity (ETI). Last, a hypersensitive response in the roots occurred, which was probably induced by the accumulation of ROS.

## Background

Members of the genus *Meloidogyne*, which represent major pests worldwide, have a substantial negative influence on the development of various plant species [1]. The J2 stage (the second stage of juveniles) is the main infection stage of RKNs; during this stage, giant cells (GCs) form in the roots of plants and absorb nutrients from the roots for their own growth and reproduction [2]. Once established, J2-stage RKNs undergo three successive molts to become adult females [3]. The development of genetic resistance is an effective method for reducing yield losses caused by RKN infection.

ROS have a substantial influence on reactions to biotic and abiotic stresses. Phytohormones such as JA and SA not only function to regulate plant growth but also are involved in plant defense signaling pathways [4]. Genes that encode nucleotide-binding site–leucine-rich repeats (NBS-LRRs) are the predominant members of the *R* gene family, accounting for approximately 80% of the more than 140 cloned *R* genes [5]. In potato, *Gpa2* is an *R* gene that encodes the GPA2 protein, which depends on the recognition specificity afforded by both amino acid 187 and the Gpa2 LRR domain and provides resistance against two *Globodera pallida* nematode populations (D383 and D372) [6]. Additionally, the *Rhg1* gene from soybean, the *Me3* and *Me4* genes from pepper, and the *Mi-1* gene from tomato provide resistance against specific strains of nematodes.

The *Mi-1* gene, which has been mapped to the short arm of chromosome 6 and whose product contains a putative coiled-coil domain preceding the nucleotide-binding site (NBS) [7], is the major effective *R* gene against RKN species in tomato (*Solanum lycopersicum*) [8]. The *Mi* genomic region contains three homologous genes, which are referred to as *Mi-1.1*, *Mi-1.2* and *Mi-1.3*. Of these genes, only *Mi-1.2* provides resistance against RKNs, including *Meloidogyne incognita*, *Meloidogyne javanica* and *Meloidogyne arenaria* [9]. *Mi-1* is an effective genetic resource for use against RKNs; however, *Mi-1*-mediated resistance is inactive at soil temperatures greater than 28 °C [10]. Thus, additional heat-stable genes that provide resistance against RKNs must be identified to overcome this barrier. Recently, *Mi-3* has attracted increased attention because of its heat-stable characteristics. In *Solanum peruvianum* LA3858, *Mi-3* is located on the short arm of chromosome 12 and contains a 600-kb contig between the *Mi-3*-flanking markers TG180 and NR18, corresponding to a genetic distance of approximately 7.2 cM [11]. Although it originates from a wild species, *Mi-3*, whose product functions effectively when temperatures reach 32 °C, could be a valuable source of resistance for cultivated tomato. However, self-incompatibility and distant hybridization incompatibility are the primary barriers preventing this gene from being finely mapped and cloned [12].

This study was designed to investigate via RNA sequencing (RNA-seq) *Mi-3*-mediated resistance in plants grown at two different soil temperatures (34 °C and 25 °C) following inoculation with *M. incognita*. Our goal was to identify key DEGs at the transcriptional level from the perspectives of PTI and ETI. We analyzed the plant defense response pathways with which these DEGs were significantly involved. Last, the *Mi-3* gene-mediated disease response was characterized via a functional analysis of the proteins encoded by the DEGs in combination with analyses of the differences in DEG expression trends between the resistant (25 °C) and susceptible (34 °C) lines.

## Results

### RKN disease evaluation under different temperature treatments

**Fig. 1:**
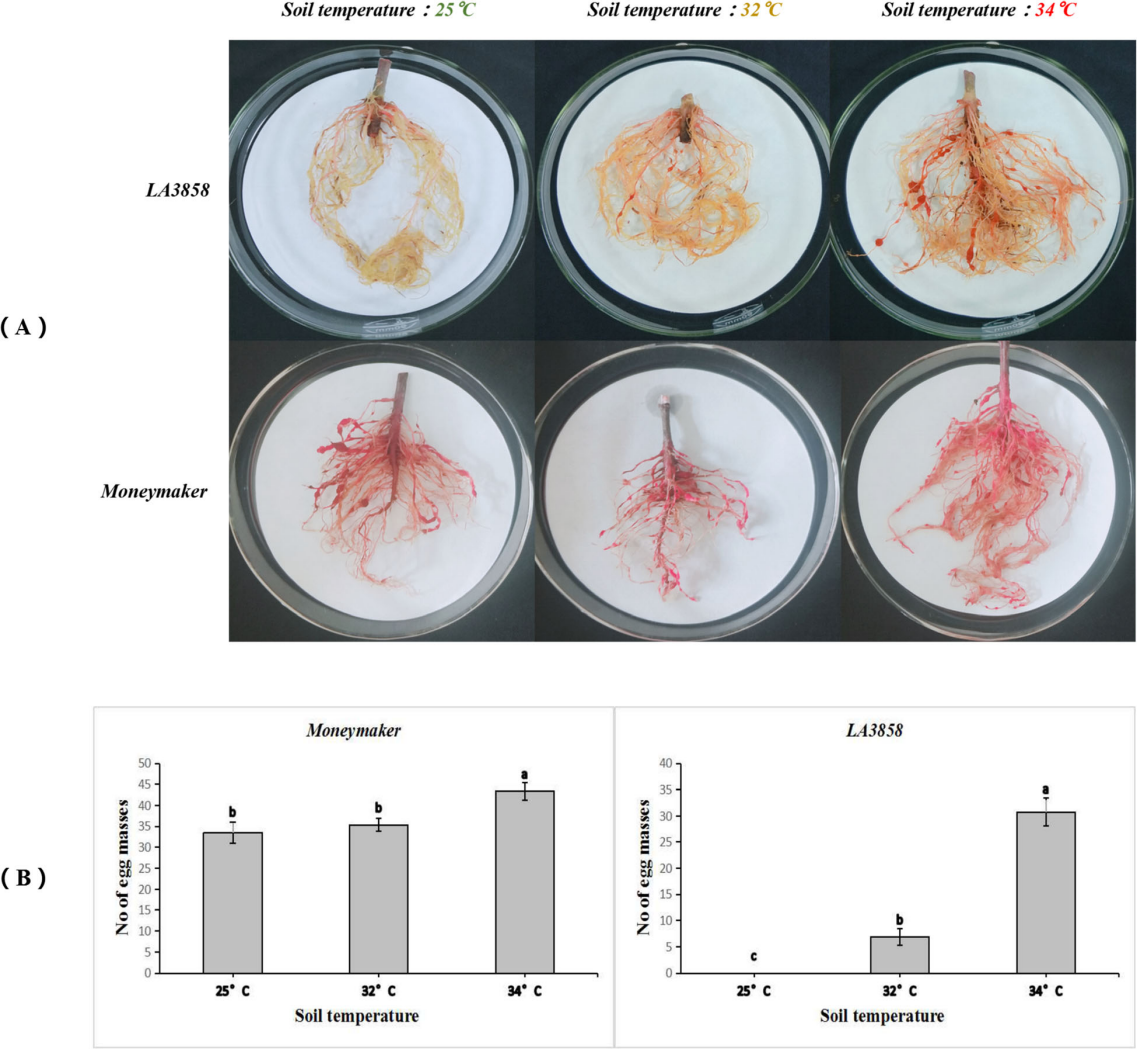
**a** Resistant phenotypes of LA3858 and Moneymaker at different soil temperatures after infestation by *M. incognita*. Seedlings of LA3858 and Moneymaker grown at 25 °C, 32 °C and 34 °C soil temperatures for five days before inoculation. At 45–50 days after infection with *M. incognita*, the roots were dyed with acid fuchsin solution, and the disease resistance was evaluated. **b** Number of egg masses on the roots of LA3858 and Moneymaker infected by nematodes. Quantities of egg masses on the roots of two different strains, which had been growing under different soil temperatures (25 °C, 32 °C and 34 °C). One-way ANOVA was performed to determine the relevant significance within the same line after inoculation

According to the infection results, the Moneymaker line displayed relatively consistent susceptibility to *M. incognita* at both soil temperatures (Fig. 1a)*.* According to the resistance index results, all the seedlings were rated as S and HS (Additional file 1). In accession LA3858, all 8 plants at the soil temperature of 25 °C were immune to infection (rated as HR). At the soil temperature of 32 °C, very small galls had developed on the roots; however, all plants were rated as HR. When the plants were subjected to a soil temperature of 34 °C, gall formation on the roots was enhanced, and the resistance was rated as S and HS. According to a one-way ANOVA, although the number of egg masses from the Moneymaker line was not significant (*P* > 0.05), the root gall numbers on accession LA3858 plants in the 34 °C soil temperature treatment were obviously greater than those on plants in the other two soil treatments (*P ≤* 0.05), which indicated that any resistance to RKNs of LA3858 will be completely absent (HS) at 34 °C (Fig. 1b).

### Illumina sequencing and alignment to the reference genome

**Fig. 2:**
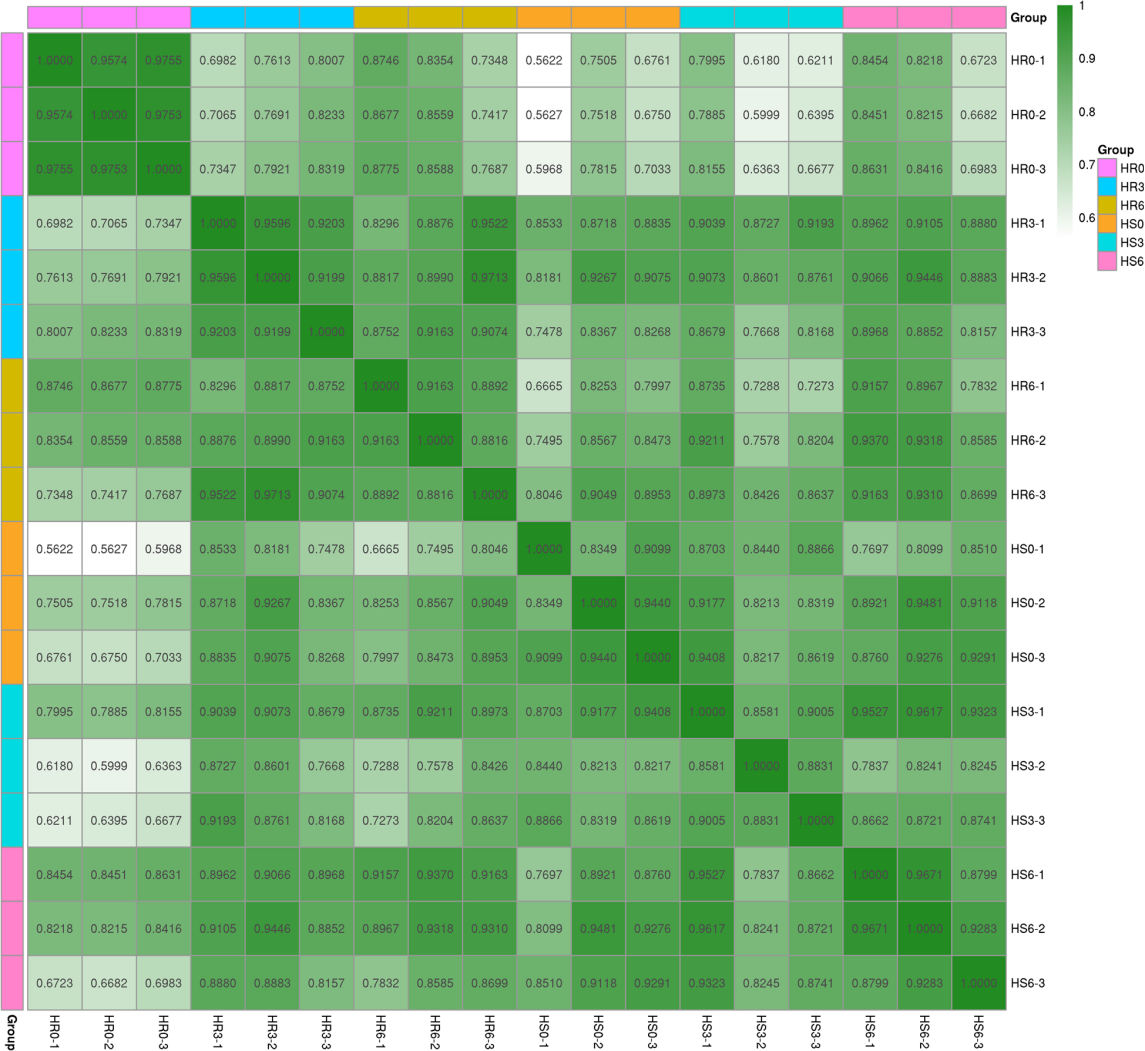
Pearson correlation coefficients of all 18 samples. The expression level of each gene (the entire gene set) for each pair of samples was used to calculate the Pearson correlation coefficients, and the correlation coefficients between the two samples were visually displayed as a heat map

RNA-seq data were generated from 18 samples of the wild species LA3858 at different stages following inoculation (0, 3 and 6 dpi). After sequencing a total of 89 billion fragments of clean reads, we obtained approximately 49 M reads for each sample after aligning them to the reference genome (SL 3.0, ftp://ftp.sgn.cornell.edu/genomes/Solanum_lycopersicum/assembly/build_3.00/) via TopHat2 (version 2.0.3.12). Approximately 46 M clean reads per sample were obtained for subsequent analysis after the rRNA sequences, adapter sequences and low-quality reads were filtered and removed. The Q20 values (base calling error probability = 99%) of the 18 samples were greater than 98%. The expression profiles of 35,768 genes were ultimately used for further analysis (Fig. 2).

### DEGs observed at 25 °C and 34 °C soil temperatures

**Fig. 3:**
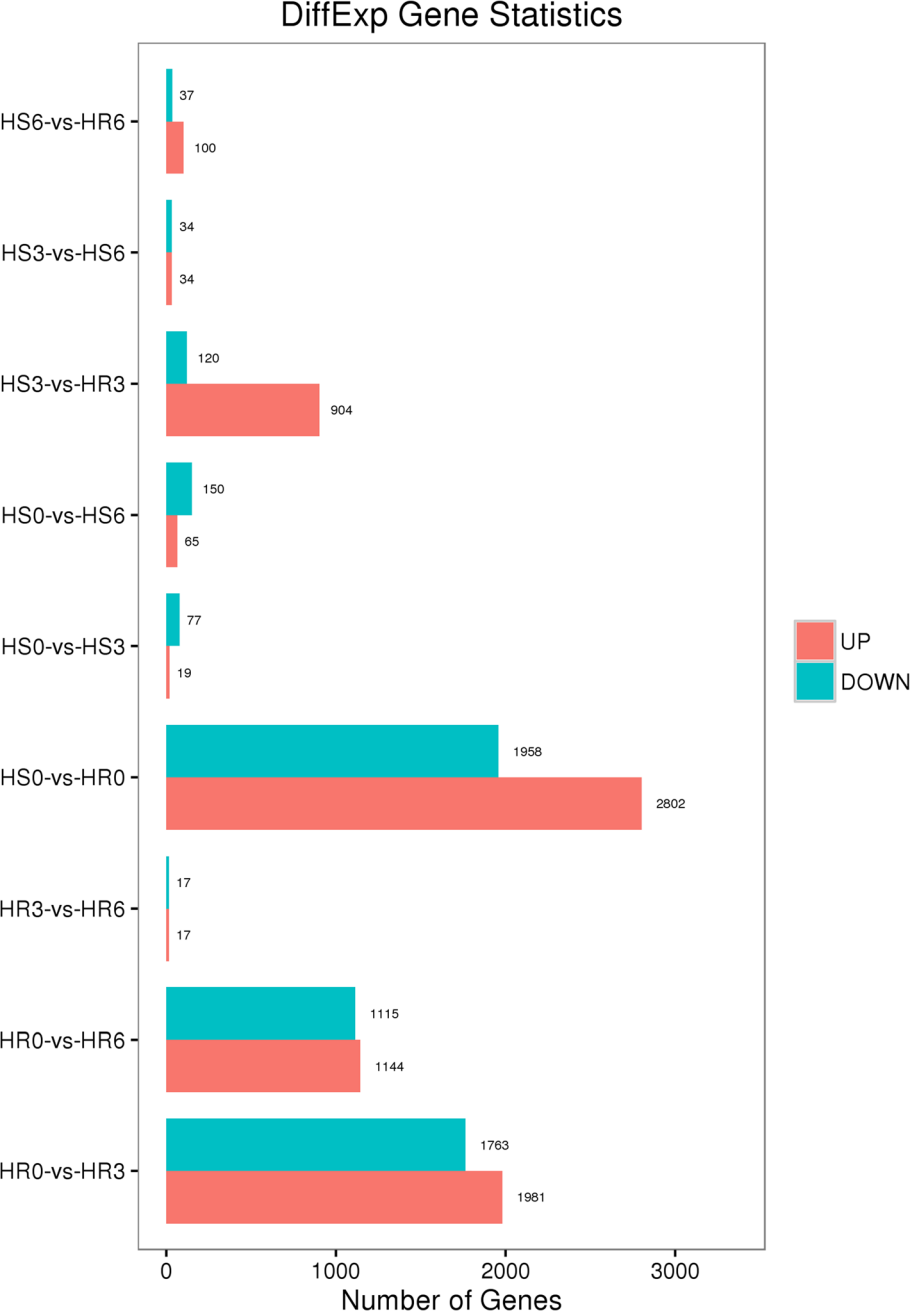
Statistics of the DEGs among different comparison groups. The FDR and log2FC were used to screen for DEGs with an FDR < 0.05 and a |log2FC| > 1

**Fig. 4:**
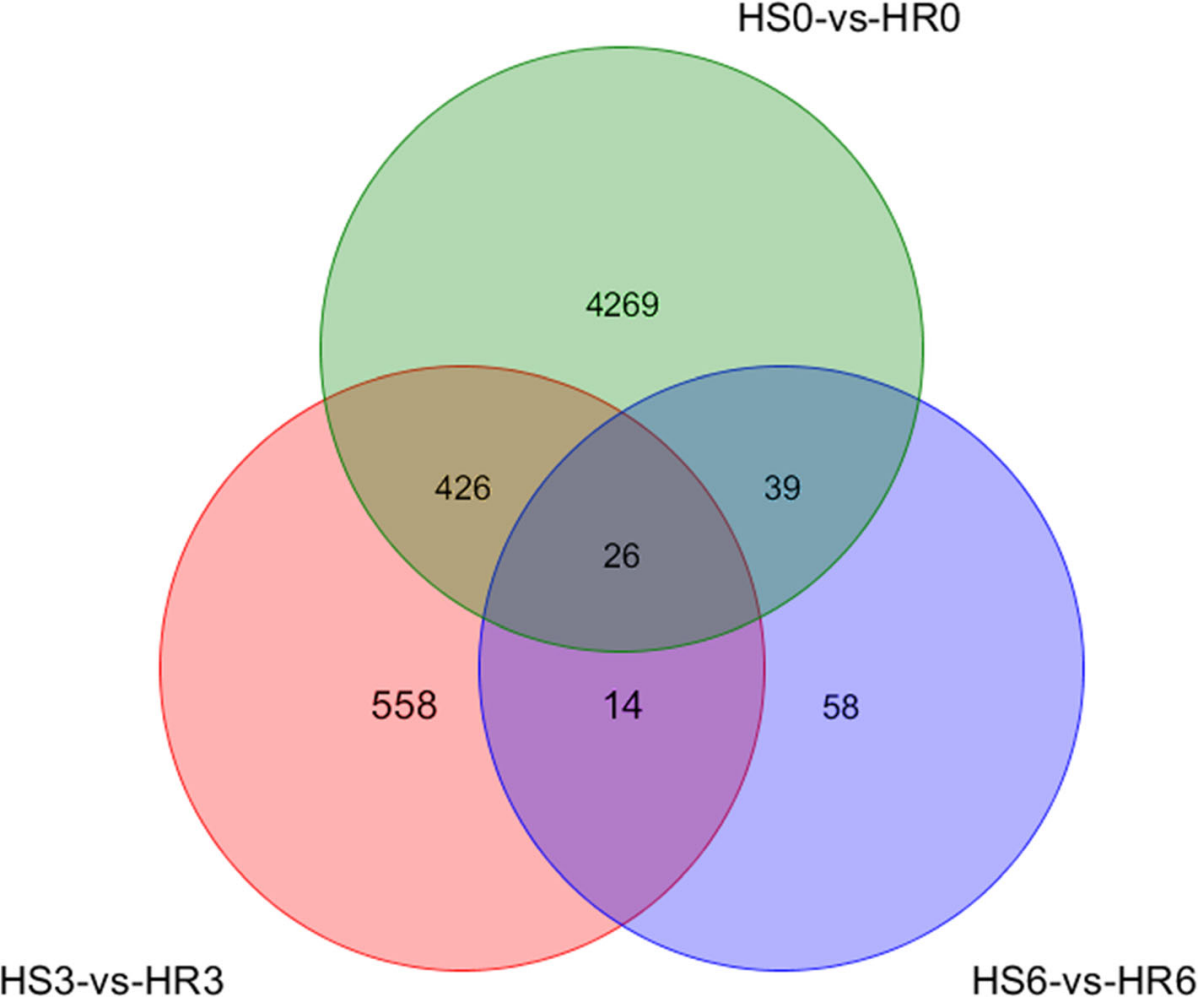
Venn diagram of the different groups of DEGs. The overlaps of DEGs from the pairwise comparisons of three groups (A – HS0 vs. HR0, B – HS3 vs. HR3, and C – HS6 vs. HR6). The DEGs were chosen at 0, 3 and 6 dpi as time points for analysis

The DEG analysis revealed that 5921 DEGs were enriched (*P ≤* 0.05) in the three groups; the expression of 2802, 904 and 100 of these genes was upregulated, and that of 1958, 120 and 37 DEGs was downregulated in 34 °C vs. 25 °C at 0 (before inoculation), 3 and 6 dpi (HS0 vs. HR0, HS3 vs. HR3 and HS6 vs. HR6), respectively (Fig. 3). The overlapping genes among these groups are shown in Fig. 4.

**Fig. 5:**
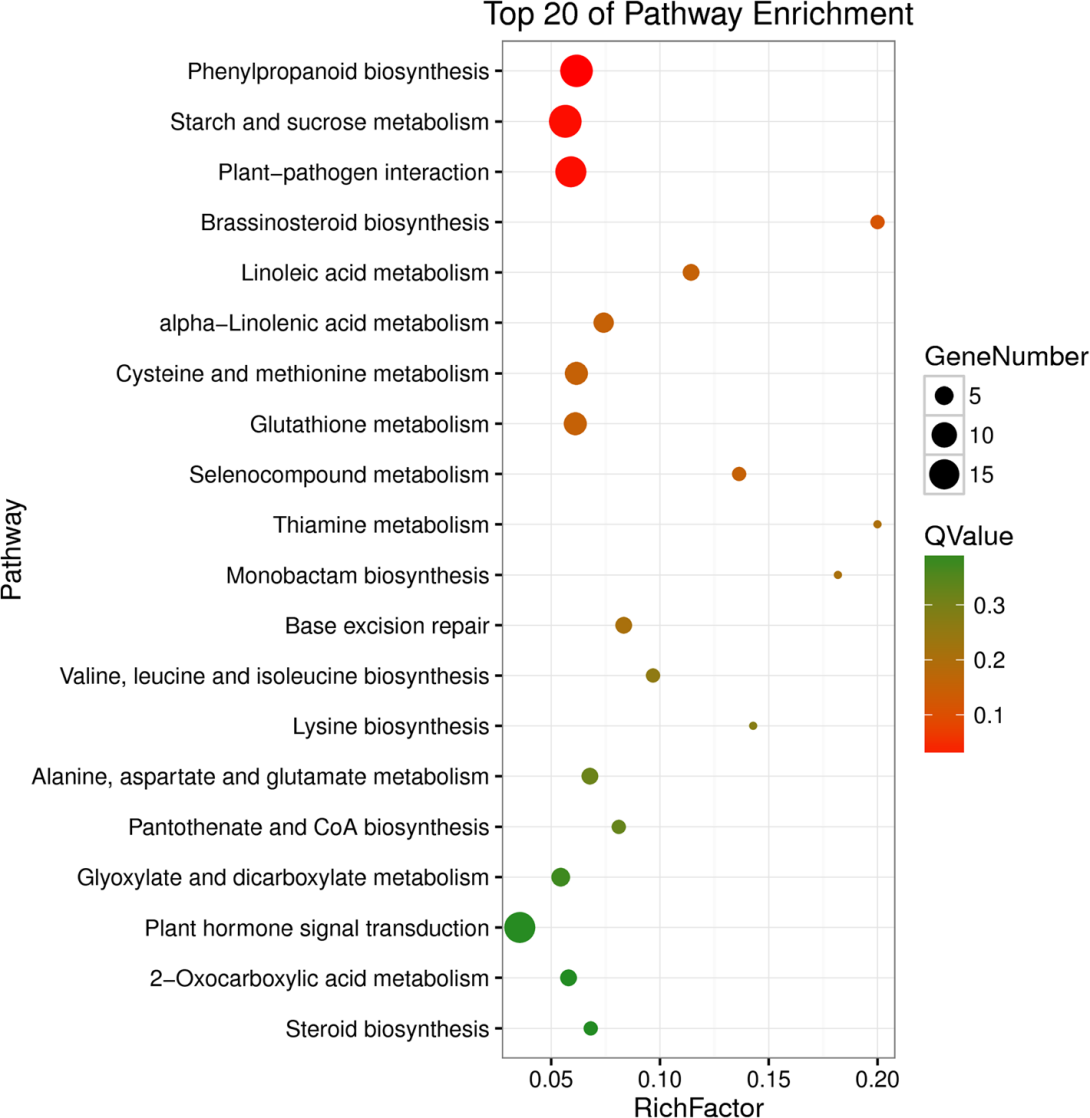
KEGG pathway analysis of pairwise comparisons (HS3 vs. HR3) at 3 dpi. KEGG pathways analysis for HS3 vs. HR3 (34 °C vs. 25 °C-3 dpi). The chart shows the top 20 pathways enriched in the selected group. The plot contains Q-values (< 0.05) shown from the smallest to the largest for the 20 pathways. The values range from 0 to 1, and the closer to 0 the value is, the more significant the enrichment

GO enrichment analysis revealed the enrichment of the DEGs with respect to the following three categories: cellular component, molecular function and biological process. Most of the DEGs were enriched (*P ≤* 0.05) in the cellular component category and were involved in the “cell”, “cell part”, “membrane”, and “organelle” terms. The significantly enriched terms (*P ≤* 0.05) in the molecular function category were “binding”, “catalytic activity” and “transporter activity”, and the enriched terms (*P ≤* 0.05) in the biological process category included “biological regulation”, “cellular process”, “metabolic process”, and “response to stimulus and signaling”, which were related to disease resistance [13] (Figure S1). Notably, in the HS3 vs. HR3 comparison, many key KEGG pathways related to biotic stress were significantly enriched (*P* ≤ 0.05), including “plant-pathogen interaction” (16 DEGs), “plant hormone signal transduction” (16 DEGs), and “brassinosteroid biosynthesis” (3) (Fig. 5). However, in the HS0 vs. HR0 and HS6 vs. HR6 comparisons, enrichment of these key pathways was not obvious.

### Trends of DEGs in the same lines (25 °C and 34 °C)

**Fig. 6:**
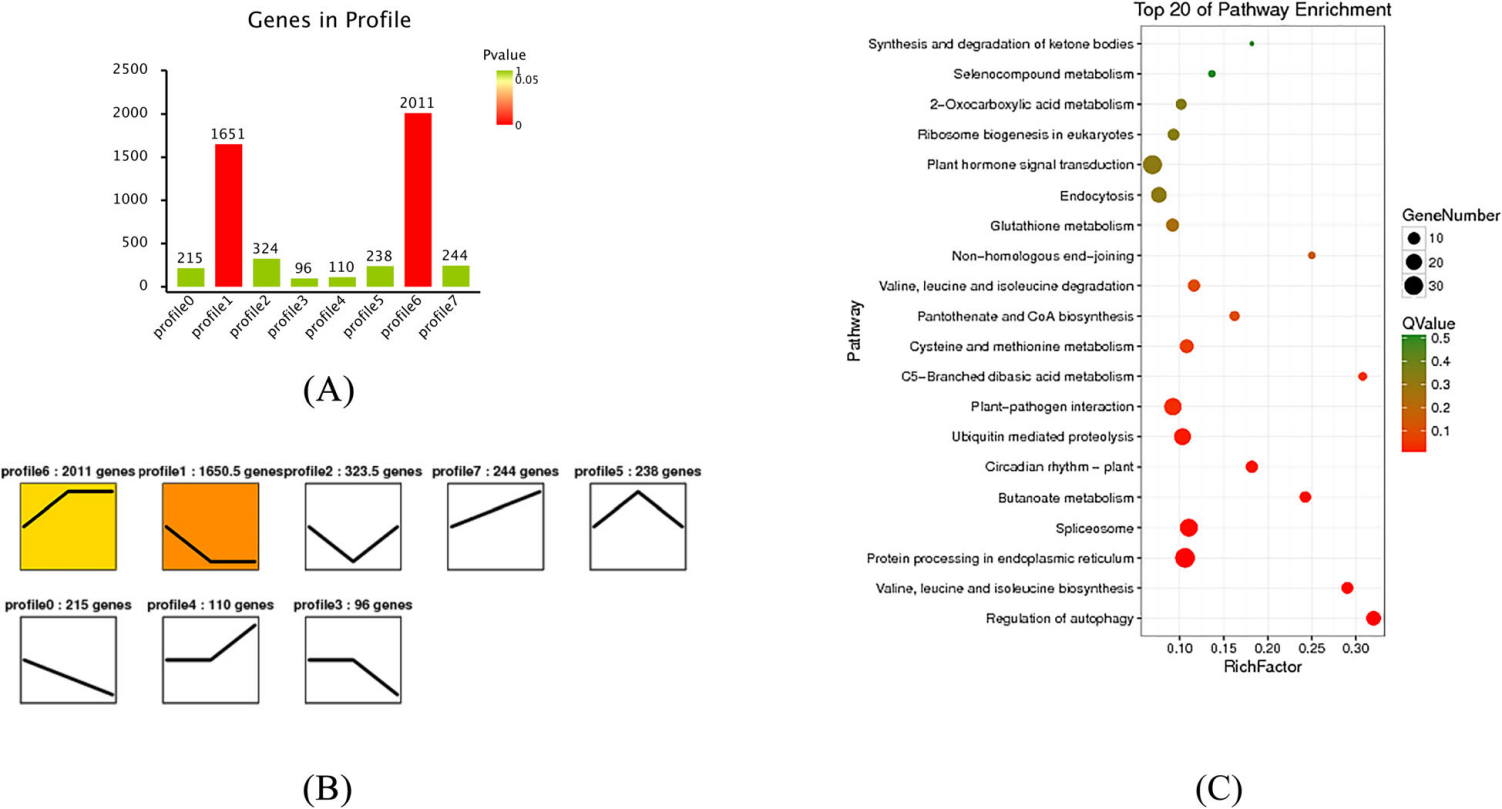
Trend analysis in 25 °C line (HR) and significantly enriched KEGG pathways. (**a**) Number of DEGs in significantly enriched (*P* ≤ 0.05) trend profiles (profile 1 and profile 6). (**b**) Unique expression trend of DEGs in profiles 1 and 6. (**c**) Top 20 significantly enriched pathways in 25 °C line

**Fig. 7:**
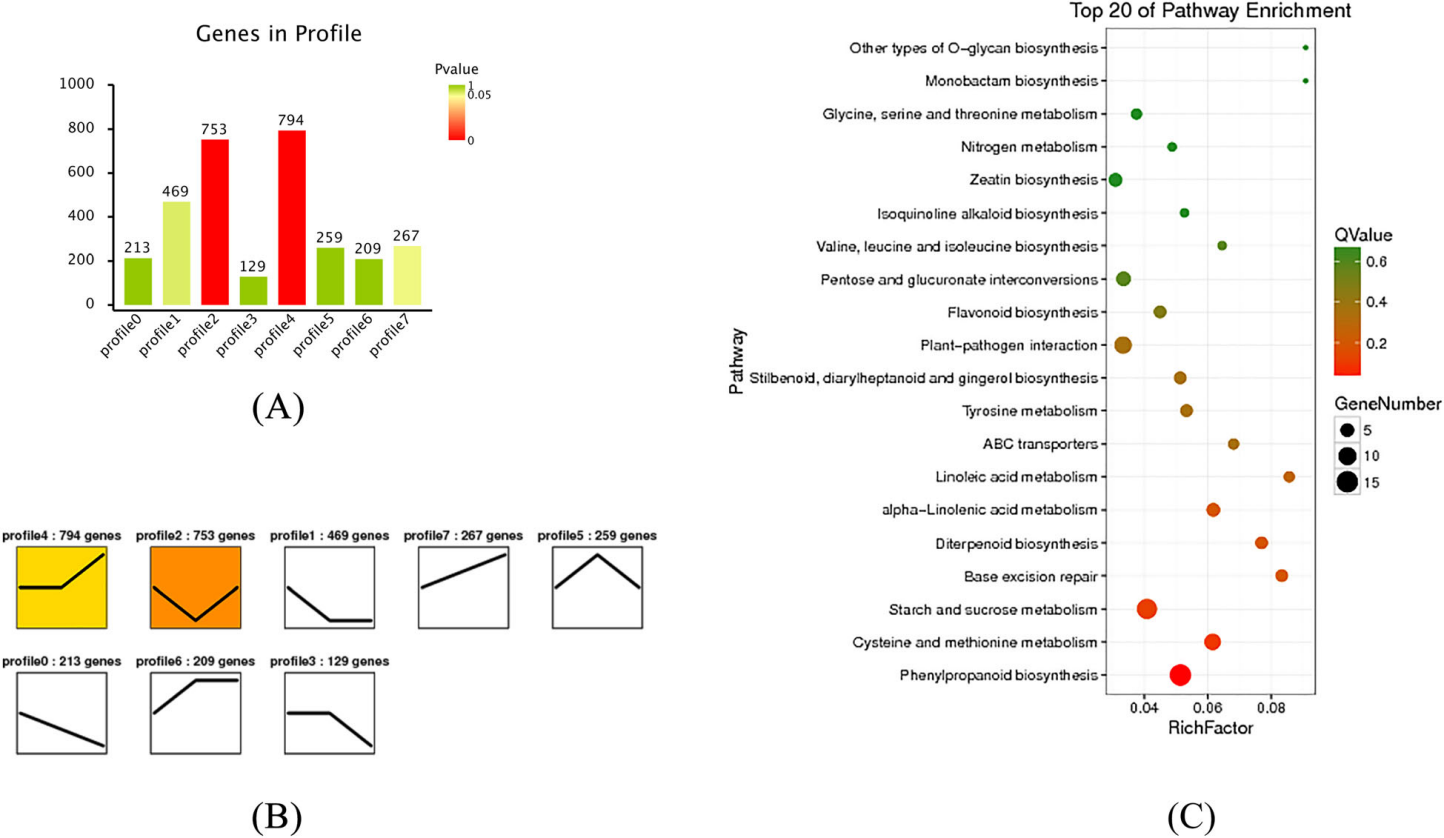
Trend analysis in 34 °C line (HS) and significantly enriched KEGG pathways. (**a**) Number of DEGs in significantly enriched (*P* ≤ 0.05) trend profiles (profile 2 and profile 4). (**b**) Unique expression trend of DEGs in profiles 2 and 4. (**c**) Top 20 significantly enriched pathways in 34 °C line

To analyze the DEGs in the same lines at different time points (0, 3 and 6 dpi), trend analysis was used to discover DEG expression patterns. When LA3858 was subjected to the 25 °C soil temperature treatment, most DEGs were enriched in profiles 1 and 6 (*P* ≤ 0.05) (Fig. 6a). The KEGG analysis revealed a unique increasing expression trend (Fig. 6b), as profile 6 exhibited a high enrichment of DEGs. Several key pathways related to disease resistance were also significantly enriched, such as “plant hormone signal transduction” (31 DEGs) and “plant-pathogen interaction” (25 DEGs) (Fig. 6c). In 34 °C line, most DEGs were significantly enriched in profiles 2 and 4 (*P* ≤ 0.05) (Fig. 7a). Similarly, because DEGs enriched in profile 2 exhibited a specific downregulation expression trend (Fig. 7b), profile 2 received increased attention. In addition to the “plant-pathogen interaction” (9 DEGs) pathway, the “phenylpropanoid biosynthesis” (15 DEGs) and “flavonoid biosynthesis” (4 DEGs) pathways, whose metabolites often play an active role in regulating the response to biotic stimulus, were also enriched in profile 2 (Fig. 7c).

### Gene expression under different temperature treatments at 3 dpi

At the important time period of 3 dpi, in terms of the key pathways, “plant-pathogen interaction” and “plant hormone signal transduction” were the primary ones identified.

In the “plant-pathogen interaction” pathway, the expression levels of 2 genes that encode calcium-dependent protein kinases (CDPKs), 3 genes that encode respiratory burst oxidases (RBOHs), and 3 genes that encode LRR receptor-like serine/threonine-protein kinases (FLS2s) were upregulated. Additionally, the expression levels of 2 genes that encode disease resistance proteins (RPs; RPM1 and PRS2) and 2 genes that encode HSPs (HSP90s) were also upregulated in the plants grown at 25 °C soil temperature. The other genes encode enhanced disease susceptibility 1 protein (EDS1), which is involved in programmed cell death (PCD), and WRKY TFs. In the “plant hormone signal transduction” pathway, 16 genes were enriched significantly (*P* ≤ 0.05), with 11 involved in the auxin response, including those encoding auxin-responsive proteins (IAAs), auxin response factors (ARFs), auxin-responsive GH3 family members and SAUR proteins; the expression of 10 of these genes was upregulated. Other genes encode a serine/threonine-protein kinase (SRK2), which is involved in the abscisic acid pathway, a brassinosteroid (BR) signaling kinase (BSK), which is involved in BR biosynthesis, and 2 TGA TFs, which are involved with SA (Table 1).

**Table 1:** Key DEGs that are involved in the resistance response to infection by RKNs and are enriched in the 34 °C vs. 25 °C at 3 dpi

Gene ID	Symbol	Log2(fold change)-HS3 vs. HR3	Pathway
Solyc01g106620.2	PR1	**−2.32**	Plant-pathogen interaction/Plant hormone signal transduction
Solyc02g072480.3	GSO1(FLS2)	**2.48**	Plant-pathogen interaction
Solyc03g033540.3	CPK16(CDPK)	**1.19**	Plant-pathogen interaction
Solyc03g044900.3	CML41	**2.11**	Plant-pathogen interaction
Solyc04g007090.1	R1A(RPM1)	**9.06**	Plant-pathogen interaction
Solyc04g014650.3	At3g47570(FLS2)	**2.43**	Plant-pathogen interaction
Solyc05g010670.3	Hsp83(HSP90)	**1.21**	Plant-pathogen interaction
Solyc06g006020.2	FLS2	**1.4**	Plant-pathogen interaction
Solyc06g036290.3	HSP83A(HSP90)	**1.42**	Plant-pathogen interaction
Solyc06g068680.3	RBOHD	**1.94**	Plant-pathogen interaction
Solyc06g071280.3	EDS1	**1.36**	Plant-pathogen interaction
Solyc07g042460.2	RBOHE	**1.66**	Plant-pathogen interaction
Solyc08g007250.2	At4g27190(RPS2)	**2.3**	Plant-pathogen interaction
Solyc08g081690.3	RBOHA	**1.42**	Plant-pathogen interaction
Solyc12g005040.2	CPK2(CDPK)	**1.53**	Plant-pathogen interaction
Solyc12g006170.2	WRKY20	**1.37**	Plant-pathogen interaction
Solyc01g096070.3	ARF9	**1.62**	Plant hormone signal transduction
Solyc01g097290.3	IAA16	**1.91**	Plant hormone signal transduction
Solyc02g092820.3	GH3.1	**−3.1**	Plant hormone signal transduction
Solyc04g081250.1	SAUR40	**10.1**	Plant hormone signal transduction
Solyc05g056550.3	SAPK7	**1.21**	Plant hormone signal transduction
Solyc06g008580.3	IAA3	**3.04**	Plant hormone signal transduction
Solyc06g074320.3	TGA21	**1.2**	Plant hormone signal transduction
Solyc06g084070.3	AUX22D	**1.71**	Plant hormone signal transduction
Solyc07g063850.3	GH3.6	**1.89**	Plant hormone signal transduction
Solyc08g008380.3	ARF9	**1.85**	Plant hormone signal transduction
Solyc08g082630.3	ARF9	**1.87**	Plant hormone signal transduction
Solyc09g083280.3	IAA4/5	**1.62**	Plant hormone signal transduction
Solyc11g068370.2	TGA2.3	**1.6**	Plant hormone signal transduction
Solyc12g096980.2	IAA13	**2.67**	Plant hormone signal transduction
Solyc12g099830.2	At5g41260(BSK)	**1.33**	Plant hormone signal transduction

The trend analysis revealed that, in 25 °C line, DEGs were enriched significantly (*P* ≤ 0.05) in profile 6 class. In the “plant-pathogen interaction” pathway; in addition to the above mentioned protein-coding genes, the genes encoding RPM1-interacting protein (RIN4) and pathogen-induced protein kinase (PIK1) were highly enriched. Compared with the 34 °C vs. 25 °C, a large number of genes were involved in other key processes, such as those associated with cytokinin (CK), gibberellin (GA), ET and JA in the “plant hormone signal transduction” pathway (Table 2). In 34 °C line, DEGs in profile 2 were highly enriched (*P* ≤ 0.05). The expression levels of 9 DEGs that encoded FLS2, RBOH, RPS2, HSP90 and WRKY, which involved into pathway “plant-pathogen interaction”, decreased at 3 dpi (Table 3).

**Table 2:** Key DEGs that are involved in the resistance response to infection by RKNs and are enriched according to the trend analysis of 25 °C line (growing at a soil temperature of 25 °C)

Gene ID	Symbol	CK-log2 (1)	log2(HR0 vs. HR3)	log2(HR0 vs. HR6)	Profile	Pathway
Solyc01g108190.3	CML1	**0**	**1.85**	**1.38**	6	Plant-pathogen interaction
Solyc02g037540.2	At4g27220(RPS2)	**0**	**1.36**	**1.44**	6	Plant-pathogen interaction
Solyc02g081040.3	CERK1	**0**	**1.12**	**1.2**	6	Plant-pathogen interaction
Solyc02g083850.3	CPK16(CDPK)	**0**	**1.38**	**1.58**	6	Plant-pathogen interaction
Solyc02g090810.3	/(CML)	**0**	**1.22**	**0.69**	6	Plant-pathogen interaction
Solyc03g005660.3	R1A(RPM1)	**0**	**0.9**	**1.21**	6	Plant-pathogen interaction
Solyc03g007250.1	CNGC1	**0**	**1**	**0.7**	6	Plant-pathogen interaction
Solyc03g007260.3	CNGC1	**0**	**1.05**	**0.9**	6	Plant-pathogen interaction
Solyc03g007890.3	HSP83A(HSP90)	**0**	**4.12**	**4.11**	6	Plant-pathogen interaction
Solyc03g026340.3	CPK26(CDPK)	**0**	**1.22**	**1.08**	6	Plant-pathogen interaction
Solyc03g033540.3	CPK16(CDPK)	**0**	**1.24**	**1.29**	6	Plant-pathogen interaction
Solyc03g044900.3	CML41	**0**	**1.5**	**0.83**	6	Plant-pathogen interaction
Solyc03g098210.3	CNGC20	**0**	**1.36**	**1.57**	6	Plant-pathogen interaction
Solyc04g007090.1	R1A(RPM1)	**0**	**1.37**	**1.44**	6	Plant-pathogen interaction
Solyc04g081910.3	CPK29(CDPK)	**0**	**1.04**	**0.65**	6	Plant-pathogen interaction
Solyc06g006020.2	FLS2	**0**	**1.18**	**1.55**	6	Plant-pathogen interaction
Solyc06g036290.3	HSP83A(HSP90)	**0**	**5.44**	**5.18**	6	Plant-pathogen interaction
Solyc06g068680.3	RBOHD	**0**	**1.21**	**0.8**	6	Plant-pathogen interaction
Solyc06g075550.3	CDL1-like (PIK1)	**0**	**1.94**	**2.52**	6	Plant-pathogen interaction
Solyc07g047960.3	WRKY1	**0**	**1.41**	**1.52**	6	Plant-pathogen interaction
Solyc07g065840.2	HSC80(HSP90)	**0**	**1.58**	**1.24**	6	Plant-pathogen interaction
Solyc08g076493.1	CML17	**0**	**1.01**	**0.71**	6	Plant-pathogen interaction
Solyc09g007020.1	PR1	**0**	**4.03**	**4.83**	6	Plant-pathogen interaction
Solyc09g059430.3	RIN4	**0**	**0.83**	**1.09**	6	Plant-pathogen interaction
Solyc12g006170.2	WRKY20	**0**	**1.37**	**1.24**	6	Plant-pathogen interaction
Solyc01g008980.3	DPBF4(ABF)	**0**	**1.48**	**1.25**	6	Plant hormone signal transduction
Solyc01g095700.3	PYL8	**0**	**1.74**	**1.42**	6	Plant hormone signal transduction
Solyc01g096810.3	EIN3	**0**	**1.03**	**0.81**	6	Plant hormone signal transduction
Solyc01g098400.3	AHP1	**0**	**0.88**	**1.04**	6	Plant hormone signal transduction
Solyc01g102300.3	PIF3	**0**	**1.03**	**0.62**	6	Plant hormone signal transduction
Solyc01g104650.3	GBF4(ABF)	**0**	**2.7**	**2.32**	6	Plant hormone signal transduction
Solyc01g107400.2	GH3.1	**0**	**1.46**	**1.02**	6	Plant hormone signal transduction
Solyc01g108087.1	ABF4	**0**	**1.87**	**1.96**	6	Plant hormone signal transduction
Solyc01g108280.3	SRK2E	**0**	**1.38**	**1.23**	6	Plant hormone signal transduction
Solyc03g120390.3	IAA17	**0**	**1.39**	**1.39**	6	Plant hormone signal transduction
Solyc03g121060.3	IAA26	**0**	**1.18**	**0.9**	6	Plant hormone signal transduction
Solyc03g121880.3	HAB1(PP2C)	**0**	**1.56**	**1.57**	6	Plant hormone signal transduction
Solyc04g012160.3	SAPK2	**0**	**1.5**	**1.39**	6	Plant hormone signal transduction
Solyc04g054320.3	TGA1A	**0**	**0.82**	**1.04**	6	Plant hormone signal transduction
Solyc04g078840.3	ABF2	**0**	**2.69**	**2.51**	6	Plant hormone signal transduction
Solyc05g014260.3	ARR11	**0**	**2.03**	**1.72**	6	Plant hormone signal transduction
Solyc05g015610.3	AHK3(CRE1)	**0**	**1.27**	**1**	6	Plant hormone signal transduction
Solyc05g052980.3	PP2CA	**0**	**1.43**	**1.49**	6	Plant hormone signal transduction
Solyc06g061180.1	PYR1	**0**	**0.98**	**1.17**	6	Plant hormone signal transduction
Solyc06g072650.1	SAUR71	**0**	**2.09**	**2.12**	6	Plant hormone signal transduction
Solyc06g074320.3	TGA21	**0**	**1.13**	**0.7**	6	Plant hormone signal transduction
Solyc06g076400.3	At2g29380(PP2C)	**0**	**2.16**	**2.53**	6	Plant hormone signal transduction
Solyc07g040990.3	Os01g0583100(PP2C)	**0**	**1.31**	**1.36**	6	Plant hormone signal transduction
Solyc08g060810.3	EBF1	**0**	**1.25**	**1.17**	6	Plant hormone signal transduction
Solyc08g082180.3	PYL9	**0**	**0.98**	**1.25**	6	Plant hormone signal transduction
Solyc09g007020.1	/(PR1)	**0**	**4.03**	**4.83**	6	Plant hormone signal transduction
Solyc10g009290.1	BHLH14(MYC2)	**0**	**2.78**	**2.43**	6	Plant hormone signal transduction
Solyc10g018340.1	SAUR36	**0**	**1.29**	**1.7**	6	Plant hormone signal transduction
Solyc10g078670.2	/(TGA)	**0**	**1.81**	**1.13**	6	Plant hormone signal transduction
Solyc12g044870.1	EIN4(ETR)	**0**	**3.2**	**1.74**	6	Plant hormone signal transduction
Solyc12g056860.2	TGA7	**0**	**1.26**	**1.19**	6	Plant hormone signal transduction

**Table 3:** Key DEGs that are involved in the resistance response to infection by RKNs and are enriched according to the trend analysis of 34 °C line (growing at a soil temperature of 34 °C)

Gene ID	Symbol	CK-log2 (1)	log2(HS0 vs. HS3)	log2(HS0 vs. HS6)	Profile	Pathway
Solyc01g099620.3	RBOHB	**0**	**−1.67**	**−0.35**	2	Plant-pathogen interaction
Solyc02g037540.2	At4g27220(RPS2)	**0**	**−1.13**	**0.13**	2	Plant-pathogen interaction
Solyc02g072393.1	At3g47570(FLS2)	**0**	**−1.98**	**−0.62**	2	Plant-pathogen interaction
Solyc02g072480.3	GSO1(FLS2)	**0**	**−1.42**	**−0.32**	2	Plant-pathogen interaction
Solyc05g010670.3	Hsp83	**0**	**−1.16**	**−0.34**	2	Plant-pathogen interaction
Solyc06g066370.3	WRKY33	**0**	**−1.72**	**−0.42**	2	Plant-pathogen interaction
Solyc06g068680.3	RBOHD	**0**	**− 0.67**	**0.38**	2	Plant-pathogen interaction
Solyc07g008620.1	/(EIX)	**0**	**−1.5**	**−0.44**	2	Plant-pathogen interaction
Solyc11g071750.2	CML37	**0**	**−10.91**	**−3.82**	2	Plant-pathogen interaction

### Analysis of the hub genes from the coexpression network during incompatible interactions

**Fig. 8:**
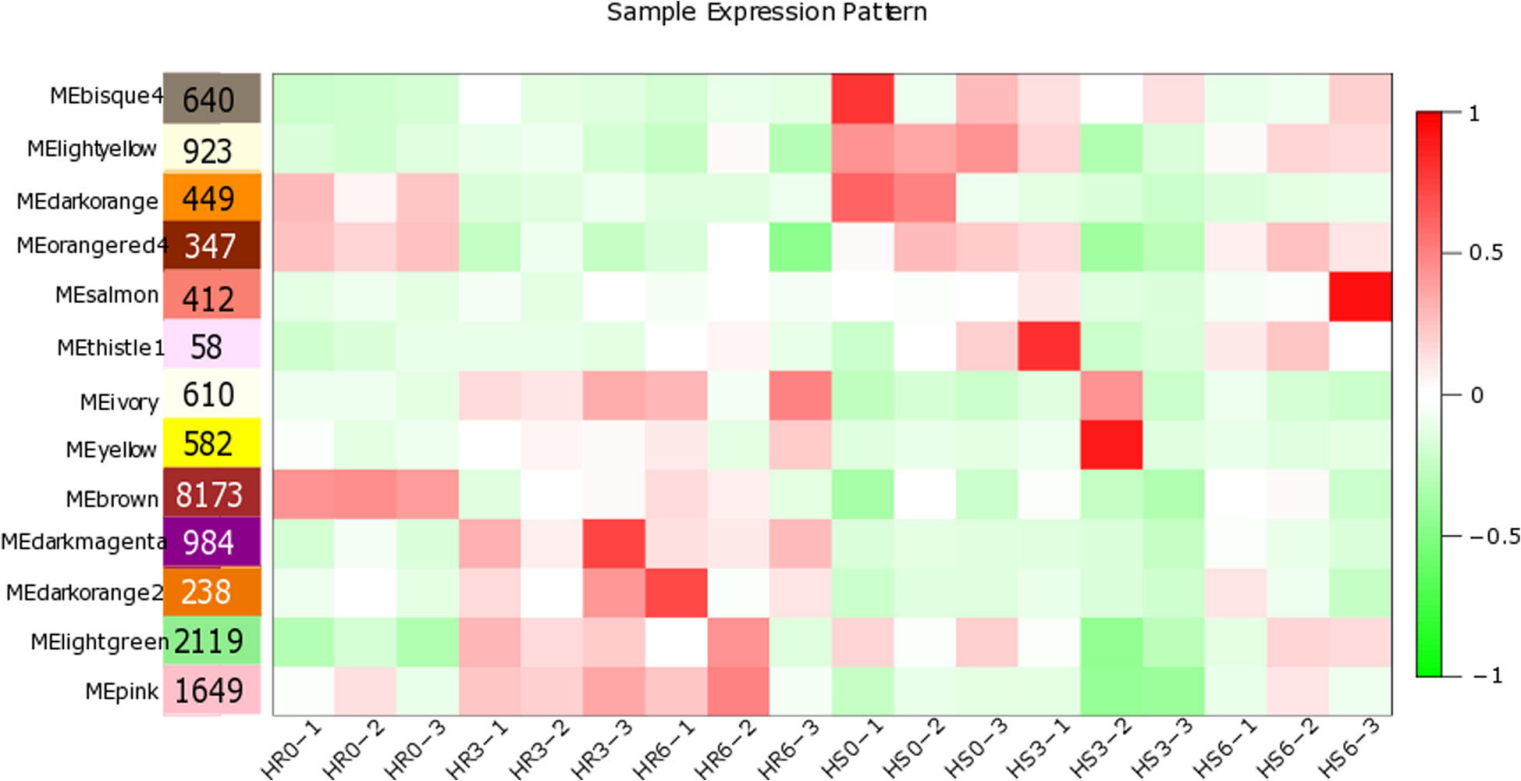
Expression pattern analysis of plant samples according to WGCNA. The expression pattern of the module genes in each sample is represented by the module eigenvalue. The abscissa represents the sample, and the ordinate represents the module, which is plotted with the module eigenvalues. The red color represents a high expression level, the green color represents a low expression level, and the number of DEGs in each module is shown

**Fig. 9:**
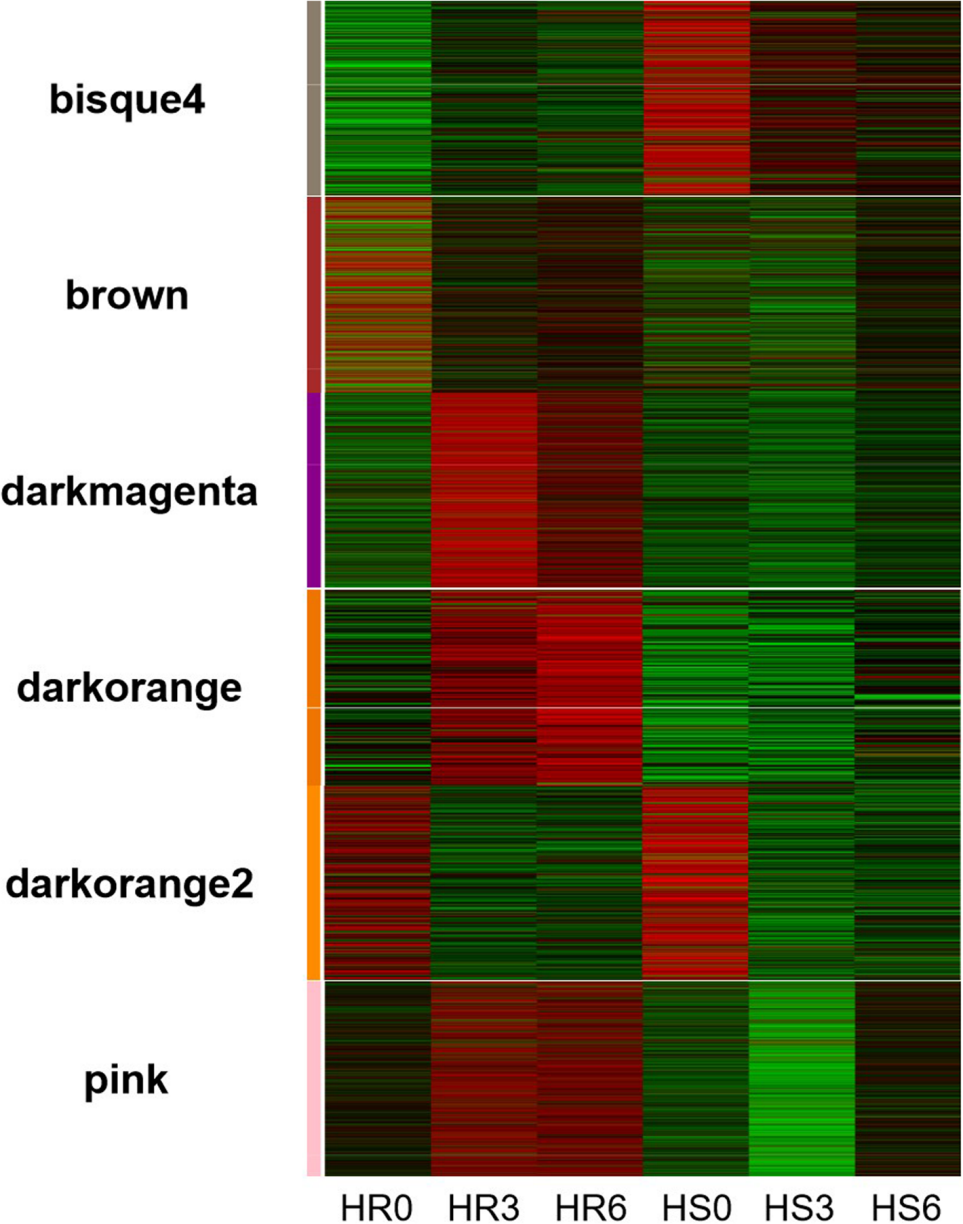
Heatmap of gene expression patterns for resistance-specific WGCNA modules. The expression pattern of each gene within each module is shown in a heat map. The left column represents the 6 modules, and the 6 vertical columns represent the 0-day, 3-day and 6-day time points from both lines (25 °C and 34 °C). Each column shows the gene expression in the different samples. The genes in the darkorange and pink modules were significantly expressed at 3 and 6 dpi and are involved in the regulation of disease resistance. The red color indicates genes whose expression level increased, and the green color indicates genes whose expression levels decreased

**Fig. 10:**
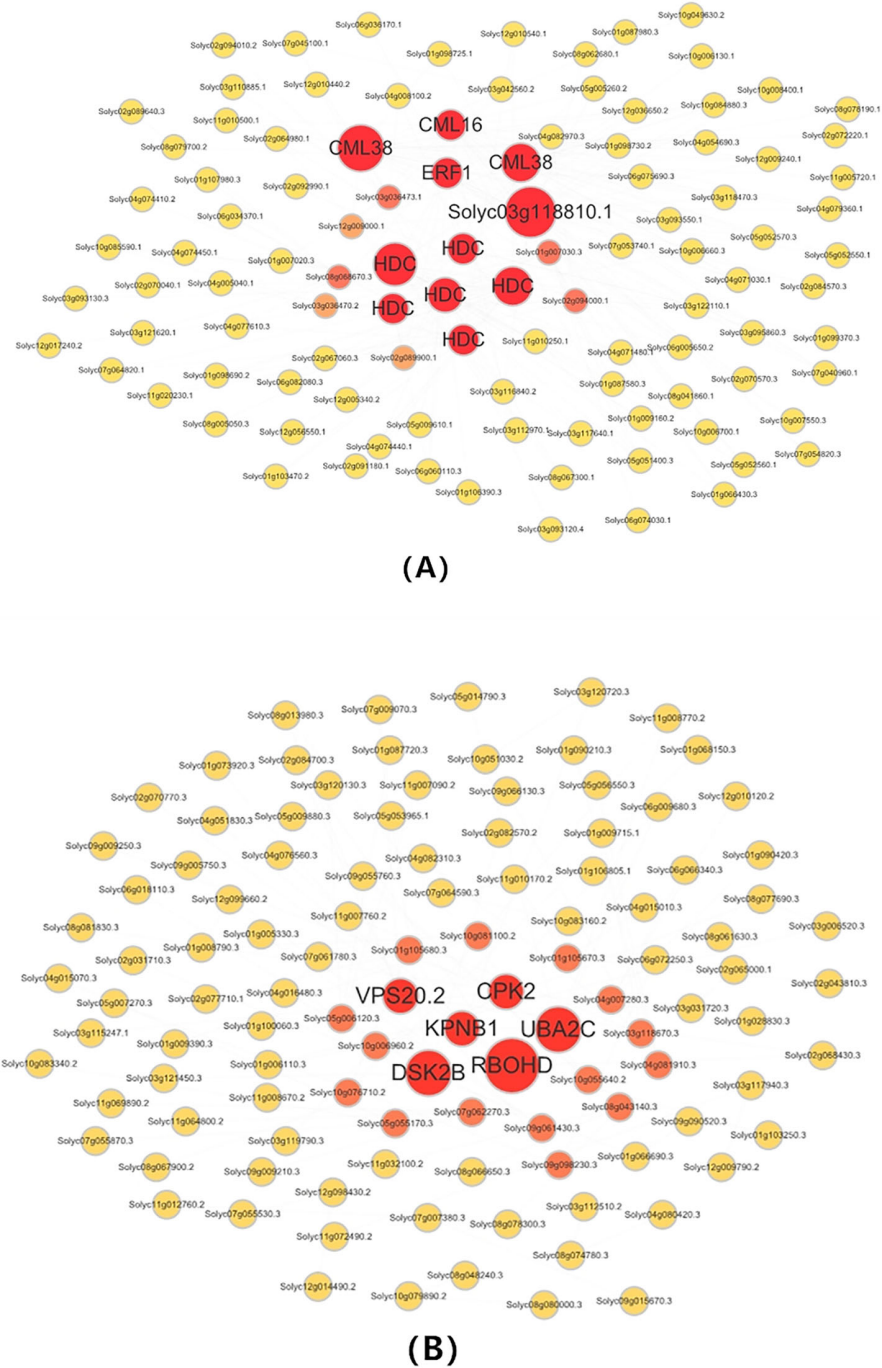
Coexpression network of the pink and darkorange modules according to WGCNA. (**a**) and (**b**) represent the darkorange and pink modules, respectively, which were filtered from the six modules by WGCNA. The red circles represent hub genes in the darkorange and pink modules, the pathway “plant-pathogen interaction” are significantly enriched

The genes related to the regulation of the resistance mechanism of LA3858 at different soil temperatures were further investigated. After clustering the module genes according to the standards mentioned above, we selected a total of 17,184 genes for the construction of a scale-free coexpression network. Thirteen coexpression modules were constructed (Fig. 8). Of these modules, a total of 6 (i.e., bisque4, brown, darkmagenta, darkorange, darkorange 2 and pink) were selected because of the specificity of the expression pattern at each time period. Among these 6 modules, which had been subjected to KEGG analysis, the darkorange and pink modules attracted our attention because the expression of the DEGs in both modules tended increase at 3 dpi (Fig. 9), and the “plant-pathogen interaction” pathway was also significantly enriched (*P* ≤ 0.05) in both modules (Figure S2). With respect to genes, Pearson correlation coefficients ≥0.8 were filtered to establish DEGs coexpression network to reveal hub genes whose expression is induced during *Mi-3*-mediated resistance (Fig. 10). In the darkorange module, 6 hub genes encode histidine decarboxylase (HDC), 3 genes encode calcium-binding protein (CML) and one encodes an ET-responsive transcription factor (ERF). In the pink module, CDPK and RBOH were also found to be encoded by hub genes. These results suggested that Ca^2+^ channels may play a key role in signal transduction during nematode infection [14], at the same time, the level of ROS may also contribute to the regulation of resistance, which is consistent with our previous results obtained from incompatible interactions [15] (Additional file 2).

### Key TFs that function during infection in *Mi-3*-mediated resistance

**Fig. 11:**
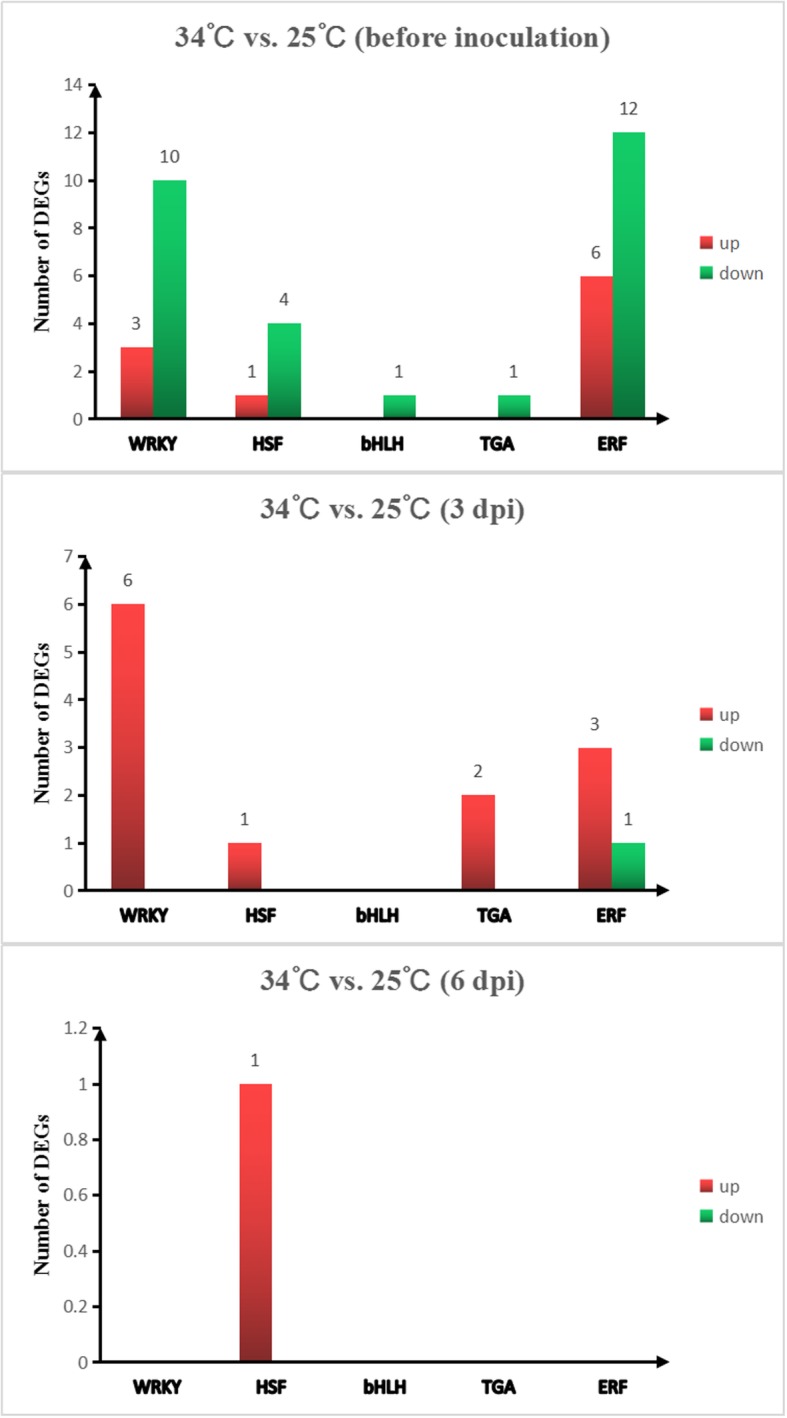
Number of WRKY, HSF, bHLH, TGA and ERF TFs significantly enriched in the 34 °C vs. 25 °C comparison at 0 (before inoculation), 3 and 6 dpi

RNA-seq analysis revealed a total of 832 TFs. Five main TF categories related to disease resistance in plants were identified: WRKYs, heat-shock transcription factors (HSFs), bHLHs, TGAs, and ERFs [16]. On the basis of the significant expression levels (*P* ≤ 0.05), we compared the detailed expression trends of the DEGs that encode TFs from the above 5 families between 34 °C and 25 °C (Additional file 3). There were more enriched DEGs that encode TFs from these families at 0 days (before inoculation) than at the other two stages (3 dpi and 6 dpi). This was most likely due to the increase in soil temperature, and the DEGs that encode TFs involved in the response to abiotic stress were significantly enriched. At 3 dpi, the expression of nearly all the significantly enriched DEGs of the TFs was upregulated, and among those that encode TFs, the number of DEGs encoding WRKY TFs was the largest. Almost no DEGs encoding TFs were enriched at 6 dpi, which indicated again that *Mi-3*-mediated resistance occurs mainly during the early stages of nematode infection (Fig. 11).

### Validation of RNA-seq data via RT-qPCR

**Fig. 12:**
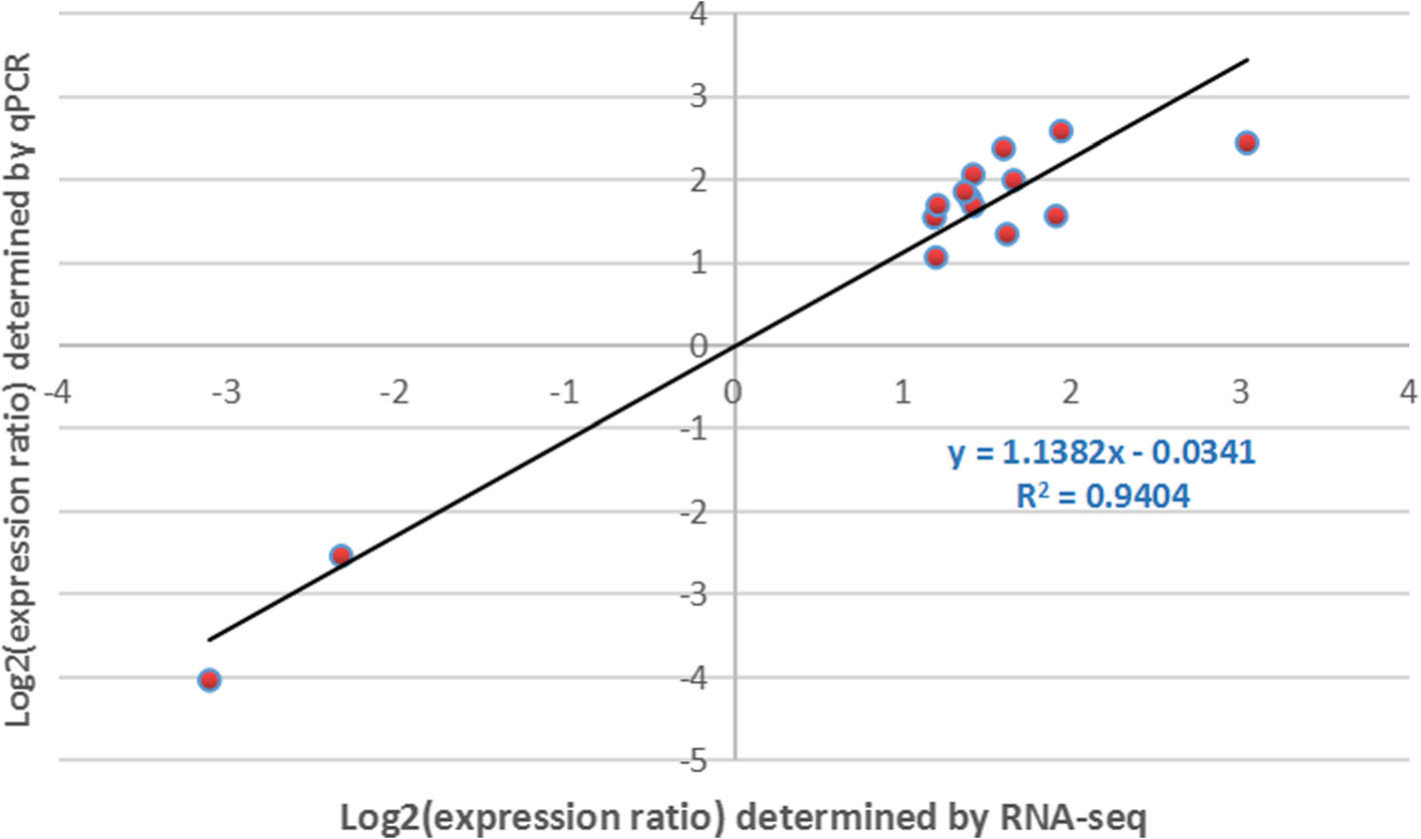
qRT-PCR validation of the RNA-seq results. Validation of gene expression (15 genes) via Pearson correlations (r values) between FCs (log2 scale) reported for the qRT-PCR results and RNA-seq results

To validate the RNA-seq results, 15 DEGs with different expression patterns were selected for qRT-PCR analysis via gene-specific primers (Additional file 4). All of these candidate genes were screened from the 34 °C vs. 25 °C comparison at 3 dpi, and there was a strong positive correlation coefficient (R^2^ = 0.9404) between the qRT-PCR results and the RNA-seq data, suggesting that the RNA-seq data were reproducible and reliable (Fig. 12).

## Discussion

In recent years, the *Mi-3* gene has attracted increased attention because of its heat-stable characteristics, as this gene remains active when soil temperatures reach 32 °C. In the present study, the results of disease resistance tests demonstrated that accession LA3858 was susceptible to *M. incognita* when the plants were growing at a soil temperature of 34 °C compared with a normal soil temperature (25 °C). We further elucidated the regulatory networks and models of the *Mi-3* gene via RNA-seq and a WGCNA (Figure S3). And RNA-seq was used to verify the transcriptomic profiles of LA3858 in response to *M. incognita*, and the reliability of the RNA-seq data was verified by the significant positive correlation detected between the qRT-PCR data and the RNA-seq data (Additional file 5). GO analysis revealed that most of the DEGs were enriched in the “cell wall”, “external encapsulating structure”, “cell division” and other key terms at 3 dpi between 34 °C and 25 °C, which may be related to GC formation [17]. DEGs were significantly enriched in each of the identified KEGG pathways, which were shown to be associated with plant immunity in a previous study. The initial stage of infection was a key period for RKN penetration and migration in tomato [18]; thus, on the basis of this timing, 3 dpi was selected as the primary stage for analysis.

### The PTI defense system is formed by *Mi-3*-mediated disease resistance

The resistance formed in response to external stimuli in plants is referred to as the plant natural immune system and can be divided into two levels, the first of which is called PTI. Plant-surface PRRs specifically recognize the conserved molecular structure of PAMPs of the pathogen, eventually inducing PTI [19]. flg22 is by far the most researched type of PAMP, which is specifically recognized by FLS2 [20]. In this work, LA3858 plants growing at 25 °C soil temperature were resistant to *M. incognita*, and compared with those in plants growing at a 34 °C soil temperature, the expression of *Solyc02g072480.3-*, *Solyc04g014650.3-* and *Solyc06g006020.2*-encoded FLS2 was upregulated at 3 dpi. And *Solyc06g006020.2* were having an increasing trend in 25 °C line (profile 6) at 3 dpi and 6 dpi; however, *Solyc02g072480.3* and another DEG (*Solyc02g072393.1*) also encoding FLS2 tended to decrease in 34 °C line (profile 2) at both 3 and 6 dpi (Tables 1, 2 and 3). This most likely indicated that in plants with *Mi-3* at 34 °C soil temperature, losing resistance was related to the obstruction of developing PTI. After PRRs recognize PAMP molecules, plants can induce rapid defense responses in a short period of time, which can involve activating mitogen-activated protein kinase (MAPK) signaling pathways, increasing ROS levels, and activating SA and JA signaling pathways [21].

### Increases in Ca^2+^ signal channels and ROS in response to J2 inoculation

After PTI develops, Ca^2+^ signal channels are instantly activated by MAPKs, and both CMLs and CDPKs are important components of these channels [22, 23]. The expression of three DEGs encoding CPK2, CPK16 and CML41 was upregulated at 3 dpi in the 34 °C vs. 25 °C comparison. Previous research has suggested that CDPKs are closely related to ROS levels during pathogen damages. RBOHs, also known as plant NADPH oxidases, are involved in the production of ROS. RBOH N-terminal domains contain two EF-hands, which are involved in calcium binding [24]. Via protein phosphorylation, RBOHs are activated to produce ROS [25]. Consistent with this function, in the present study, RBOHs were encoded by 3 DEGs whose expression was upregulated at 25 °C compared with 34 °C at 3 dpi. Therein, *Solyc06g068680.3*, which encodes RBOHD, was also significantly enriched (*P* ≤ 0.05) in 25 °C line according to the results of the trend analysis (profile 6), and its expression was downregulated in 34 °C line (profile 2) at both 3 and 6 dpi (Tables 1, 2 and 3). It was also found that this DEG is a hub gene in the pink module and that its regulates the production of ROS together with that of *Solyc12g005030.2*, which encodes another CDPK (Additional file 2). Apparently, RBOHD-dependent ROS production may play a positive role during *M. incognita* infection. According to a previous study, early production of ROS could be a key biological barrier against disease progression in plant development [26]. In the process of *Mi-3*-mediated resistance, we speculate that, owing to the rapid accumulation of ROS, *M. incognita* at the J2 stage cannot survive the relatively high oxygen concentration in the environment. Furthermore, cell damage and PCD resulting from ROS may be two reasons why nematode feeding sites (NFSs) could not be established, which prevented nutrient extraction and prevented RKNs from forming GCs [3].

### Differential expression of WRKY TFs between 34 °C and 25 °C soil temperatures

When Ca^2+^ signal channels are instantaneously activated, in addition to ROS levels, the expression of downstream TFs is also regulated [27]. The results indicated that the expression of many more TFs whose encoding genes were significantly enriched was upregulated at 3 dpi. WRKY TFs are extremely important in plant growth and disease resistance and are widely studied in tomato [28]. The expression of six key genes encoding WRKY TFs was significantly upregulated at 25 °C compared with 34 °C at 3 dpi. Among these TF genes, the expression of *Solyc04g051540.3*, *Solyc12g006170.2*, *Solyc10g009550.3*, *Solyc08g067340.3, Solyc01g095630.3* and *Solyc03g095770.3*, which encode WRKY13, 20, 30, 40, 41 and 70, respectively, differed by approximately 5.10-, 2.58-, 9.23-, 4.70-, 3.46- and 4.91-fold (Additional file 3). In addition to WKRY20, which was most likely related to drought tolerance [29], other five WRKYs all might contribute to plants disease defense. In a previous study, *WRKY13* was shown to mediate disease resistance to RKNs by activating SA-dependent pathways and by suppressing JA-dependent pathways in rice [30]. Overexpression of *WRKY30* in rice increased resistance to rice sheath blight fungus by upregulating the expression of the JA synthesis-related genes *LOX* and *AOS2* and a series of pathogenesis-related (*PR*) genes [31]. The expression of *GmWRKY40* was strongly induced in soybean following infection with *Phytophthora sojae*, and silencing *GmWRKY40* was shown to increase susceptibility [32]. WRKY41 is likely to be a key regulator in the cross talk of SA and JA pathways. The overexpression of *WRKY41* leads to an increasing of *PR5* expression, for enhancing the resistance to *Pto* wild-type [33]. Other studies have shown that, in tomato, silencing of *SlWRKY70* attenuates *Mi-1*-mediated resistance against RKNs [34]. In short, WRKYs are often involved in various defense mechanisms associated with plant growth and stress responses. Despite their involvement in the regulation of expression of some key *PR* genes and *R* genes, many WRKYs may also contribute to JA- and SA-defense signaling processes [35, 36].

### Phytohormone regulation during *M. incognita* infection

Phytohormones have simple molecular structures and low cellular levels [16]. SA, JA and ET play crucial roles in resistance to biotrophic and necrotrophic pathogens, and TGAs, MYC2 and ERFs are key TFs involved in the biosynthesis of the above three phytohormones [37]. In this research, *Solyc10g009290.1* was found to encode MYC2, which, as a member of the basic helix-loop-helix (bHLH) TF family, controls JA-dependent responses and was highly expressed in 25 °C line (profile 6). 2 and 3 DEGs encoding TGA and ERF TFs, respectively, were highly expressed at 3 dpi between 34 °C and 25 °C. Moreover, the expression of several DEGs that encoded ethylene receptors (ETRs), EIN3-binding F-box proteins (EBF1/2), and ethylene-insensitive protein 3 (EIN3) was upregulated in 25 °C line (profile 6), which contributed to the biosynthesis of the ET (Table 2). In addition, *Solyc03g093610.1*, which encodes the ET-responsive TF (ERF1), was a hub gene in the darkorange module (Additional file 2). Previous reports have shown that obstacles to the synthesis or accumulation of SA result in consistent increases in the number of phenotypes associated with susceptibility to soybean cyst nematode (SCN) [38]. JA can reduce the damage caused by RKNs, and a recent report revealed that, in JA-overexpressing transgenic tomato plants, proteinase inhibitor II (PI-II) translation levels gradually increased with time after inoculation with RKN; this increase was related to the production of JA, which acts at the initial site of infection, inhibiting nematode invasion and propagation [39]. ERF1 integrates defense signals from the ET and JA pathways and induces the expression of downstream defense-related genes [40]. In short, SA and JA defense signaling processes are important components of the first-layer defense mechanism of plants after the development of PTI [38]. According to previous research, both signaling pathways are likely to be involved in *Mi-3*-mediated resistance to *M. incognita*.

### Roles of induced disease resistance genes related to *Mi-3*

When a host plant recognizes a pathogen and produces an immune response, the pathogen will produce a substance—an effector—to inhibit the host’s recognition. To prevent additional damage, plants directly or indirectly recognize effectors via R proteins, and initiate a rapid and violent hypersensitive response at the infection site, which constitutes the second level of ETI [19]. Most *R* genes encode proteins that include a unique NBS-LRR domain. Members of the NBS-LRR protein family generally participate in downstream signal transduction during plant-pathogen interactions [41].

Two genes (*Solyc08g007250.2* and *Solyc04g007090.1*) were found to encode the disease RPs RPS2 and RPM1, which attracted our attention because of their significant increase (*P* ≤ 0.05) in expression at 25 °C compared with 34 °C at 3 dpi. It was suggested that both RPM1 and RPS2 are members of the NBS-LRR protein family. A previous report showed that these two R proteins recognize the AvrRpm1 type III effector avirulence protein involved in protecting plants against pathogens [42]. The *Solyc04g007090.1* gene encodes RPM1 and exhibited 2.58- and 2.71-FCs in expression levels in 25 °C line (profile 6) at 3 and 6 dpi, respectively; this gene was not obviously enriched in 34 °C line (profile 2). In addition, the gene *Solyc02g037540.2*, which encodes another RPS2, showed 2.57- and 2.71-fold increases in 25 °C line (profile 6) at 3 and 6 dpi, respectively. And showed downward trend in 34 °C line (profile 2). Therefore, RPM1 and RPS2 may be key proteins during infection. Additionally, *Solyc09g059430.3* was found to encode RIN4, a negative regulator of basal defense responses, whose expression was upregulated in 25 °C line (profile 6) along with that of PRS2 and RPM1 (Tables 1, 2 and 3). The biological functions of these two R proteins are associated with RIN4 [43]. RIN4 physically interacts with RPM1 and indirectly interacts with RPS2. Moreover, the RPM1 and RPS2 proteins work together to maintain RIN4 expression levels during pathogen infection [44]. However, the biological mechanisms of these three proteins, which are regulated by *Mi-3*, in response to RKNs require further study.

### HSPs may play a positive role against nematode infection

In most cases, disease resistance mediated by both RPS2 and RPM1 is associated with RAR1, SGT1 and HSPs. RAR1 interacts with the N-terminal half of HSP90, which contains an ATPase domain, and HSP90 specifically interacts with SGT1. In *Arabidopsis*, the interaction of these three proteins regulates the stability and function of RPM1, which is an HSP-client protein [45]. In this study, the expression of *Solyc05g010670.3* and *Solyc06g036290.3*, each of which encodes an HSP90 (Hsp83 and HSP83A), was upregulated at 25 °C compared with 34 °C at 3 dpi; therein, the expression of *Solyc05g010670.3* obviously tended to decrease in 34 °C line (profile 2), and *Solyc06g036290.3* had a significant increasing trend in 25 °C line (profile 6) at 3 and 6 dpi (Tables 1, 2 and 3). Additionally, another 2 DEGs were found to encode HSP90 (HSP83A, HSC80), and the expression both tended to increase in 25 °C line (profile 6). According to a previous study, genes that encode certain HSPs activated by HSFs perceive biotic stress signals via Ca^2+^ channels, which are highly expressed at the soybean *Rhg1* locus and are involved in resistance to SCN [46]. In addition, silencing of tomato HSP90 and SGT1 led to a reduction in Mi-1 protein levels, which reduced resistance to *M. incognita* [47], demonstrating that HSP90 contributes to the resistance process in plants and that DEGs encoding HSPs in our study might have a positive effect during infection of LA3858 by nematodes. Moreover, the production of HSP can also used as a standard for detecting levels of ROS in plants [48, 49]**.**

## Conclusion

In summary, this is the first report on the resistance and susceptibility of *Mi-3* at the transcriptional level under different soil temperatures. A soil temperature of 32 °C is likely to be limiting for *Mi-3* activity, which is consistent with the previous research. When the soil temperature was less than 32 °C, after *M. incognita* infected the roots, a rapid response (PTI) occurred, and downstream signal transduction was most likely triggered through Ca^2+^ channels via MAPKs and other signaling pathway components. Key defense-related TFs were subsequently triggered, such as HSFs, TGAs, ERFs, bHLHs and WRKYs, which activated disease RPs and downstream defense pathways, such as the SA, JA and ET pathways. Notably, HSPs likely associated with R proteins such as RPM1 and RPS2 in the development of ETI during infection. Last, the formation of a hypersensitive response in the roots was likely induced by ROS, resulting in cell damage and PCD. These actions prevented the establishment of root NFSs and might constitute a key mechanism for specific resistance to *M. incognita*.

## Methods

### Plant growth conditions and nematode assays

Seeds of *S. peruvianum* accession LA3858 were obtained from the Institute of Vegetables and Flowers, Chinese Academy of Agricultural Sciences, and seeds of the susceptible breeding line Moneymaker were obtained from the Northeast Agricultural University Tomato Research Institute. At 20–25 days after the seeds were sown in plots, when the seedlings reached a height of 12–15 cm (the second-leaf stage), they were transplanted into 10-cm-diameter plastic pots that each contained 50% turf soil and 50% roseite. The plants were maintained at a 24 ± 2 °C temperature, at 60% relative humidity and under a 14-h light/10-h dark photoperiod [50]. The *M. incognita* strain was obtained from the Institute of Vegetables and Flowers, Chinese Academy of Agricultural Sciences. We obtained infected roots and stripped the egg masses, which were cleaned with 1% NaOCl, after which the eggs were hatched in distilled water to obtain J2-stage individuals for inoculation [51].

### Heat treatment for phenotypic identification and transcriptomic analysis

Approximately 60 plants of accession LA3858 and Moneymaker were grown at different soil temperatures (25 °C, 32 °C and 34 °C) for five days before inoculation. Afterward, 8 plants of both lines in each treatment were randomly picked for gall and egg mass counting 45–50 days after inoculation with 2000 J2-stage *M. incognita* nematodes per plant. For transcriptomic analysis of accession LA3858, high-temperature assays were performed in a greenhouse in which the soil temperature was greater than 32 °C (34 °C) five days before inoculation. In the other group, the plants were grown at normal temperature (25 °C). Afterward, the roots of plants from the two groups were collected at 0 days (before inoculation) and at 3 and 6 days post-inoculation (dpi) with 2000 J2-stage *M. incognita* nematodes per plant [52]. At each time point, the roots of three replicates were collected. For each biological replicate, inoculated tissue was collected from 3 random plants, and the tissue samples were pooled together to obtain sufficient root tissue materials for RNA-seq and qRT-PCR verification. All root tissue samples were stored in liquid nitrogen and then transferred to − 80 °C conditions.

### Disease score on root systems

The roots were removed from the soil, washed for several minutes, cleaned with a NaOCl solution, soaked in distilled water for 15 min, and then dyed with acid fuchsin. Finally, the galls and egg masses were counted under a microscope to assess the resistance. The root gall index (percentage of roots with galls) and the resistance index (rated according to the root gall index) were the standards used for evaluating disease resistance, and each was divided into 6 levels as follows: 0 – no galls, 1–1-10% of roots with galls, 2–10-20%, 3–20-50%, 4–50-80%, and 5–80-100% for the former and immune (I), highly resistant (HR), resistant (R), moderately resistant (MR), susceptible (S), and highly susceptible (HS) for the latter [53].

### mRNA library construction and sequencing

In total, eighteen independent mRNA libraries from the roots of LA3858 plants in 2 treatments (25 °C soil temperature and 34 °C soil temperature) and at three time points (0 days, 3 days, 6 days) with three biological replicates per treatment were sequenced [54]. After the total RNA was isolated via TRIzol™ reagent (No: 15596026, Thermo Fisher Scientific), oligo (dT) beads were used to enrich the eukaryotic mRNA, while prokaryotic mRNA was enriched by the removal of rRNA with a Ribo-Zero™ Magnetic Kit (Epicentre). The enriched mRNA was then fragmented into short fragments by fragmentation buffer and reverse transcribed into cDNA with random primers. Second-strand cDNA was synthesized with DNA polymerase I, RNase H, dNTPs and buffer. The cDNA fragments were then purified with a QiaQuick PCR Extraction Kit, end repaired, polyadenylated and then ligated to Illumina sequencing adapters. The appropriate sizes of the ligation products were selected via agarose gel electrophoresis, amplified by PCR, and sequenced by an Illumina HiSeq™ 2500 instrument by Gene Denovo Biotechnology Co. (Guangzhou, China). All the raw read data have been deposited into the NCBI Sequence Read Archive database (PRJNA494774).

### Analysis of DEGs

To identify DEGs across the 18 samples, the edgeR package (http://www.rproject.org/) was used. We considered genes significantly differentially expressed when their log_2_|fold change (FC)| was > 1 and their false discovery rate (FDR) was < 0.05 in a particular comparison [55, 56]. Gene expression pattern analysis was then used to cluster genes with similar expression patterns for multiple samples (at least 3 at a specific time point, space, or treatment dose). To examine the expression pattern of the DEGs, the expression data for each sample (in the order of treatment) were normalized to 0, log2(v1/v0) transformed, and log2(v2/v0) transformed, after which they were clustered via Short Time-series Expression Miner (STEM) software [57]. The clustered profiles with *P*-values ≤0.05 were considered significant. The DEGs in the profiles were then subjected to Gene Ontology (GO) functional analysis and KEGG pathway enrichment analysis. In this article, trend analysis was performed by clustering gene expression patterns of the characteristics of multiple continuous samples (at least 3). The gene sets that met certain biological characteristics were then selected from the clustering results. And trend analysis was together used to discuss DEGs that are in the same line and are associated with pairwise comparisons.

### Weighted gene coexpression network analysis (WGCNA)

WGCNA is a systems biology method for describing correlation patterns among genes across multiple samples. Genes that express similar patterns can be clustered and analyzed for associations between modules and specific traits or phenotypes. In this study, coexpression networks were constructed via the WGCNA (v1.47) package in R [58]. After the low-quality samples and samples that had an unstable effect on the results (genes that were not expressed in more than half of the samples and samples in which more than half of the genes were not expressed) were removed, the gene expression values were subjected to a WGCNA to construct coexpression modules via the automatic network construction function blockwiseModules with the default settings, with the exceptions that the power was 6, the TOMType was unsigned, the mergeCutHeight was 0.8, and the minModuleSize was 50 [59, 60].

To identify biologically significant modules, eigengenes were used to calculate the correlation coefficients with samples or sample traits. The intramodular connectivity (function softConnectivity) of each gene was calculated, and genes with a high connectivity tended to be hub genes (q. weighted < 0.01 as a cutoff) [61], which may act as key factors that regulate a large subset of genes to perform biological functions together. The networks were subsequently visualized by Cytoscape 3.3.0.

### qRT-PCR and validation of RNA-seq results

qRT-PCR was performed to validate the accuracy of the RNA-seq results. First-strand cDNA was synthesized via a RevertAid First Strand cDNA Synthesis Kit (K1621) from Thermo Scientific. Each sample included three biological replicates. We used the NCBI BLAST program to design the primers used for the unigenes. Relative quantitative data were calculated according to the ΔΔCT method: normalization (ΔCT = CT (sample) – CT (GAPDH)); ΔΔCT = ΔCT (sample A) -ΔCT (sample B); relative quantification = 2^-ΔΔCT^ [62].

## Supplementary information

**Additional file 1 **Disease resistance statistics of LA3858 and Moneymaker infected with *M. incognita* after treatment with different soil temperatures.

**Additional file 2.** List of hub genes filtered from darkorange and pink modules by WGCNA.

**Additional file 3.** List of transcription factors significantly enriched in the 34 °C vs. 25 °C comparison at 0 (before inoculation), 3 and 6 dpi.

**Additional file 4.** List of qRT-PCR primers used in this study.

**Additional file 5.** Verification of the correlation results between the RNA-seq and qRT-PCR data

**Additional file 6: Figure S1** Level 2 GO terms identified for DEGs in 34 °C vs. 25 °C comparison at 0 (before inoculation), 3 and 6 dpi.

**Additional file 7: Figure S2** Top 20 significantly enriched pathways in the darkorange and pink modules according to WGCNA.

**Additional file 8: Figure S3** Description of all the analyses performed in this work.

## Data Availability

We have deposited our data in Sequence Read Archive (SRA) (http://www.ncbi.nlm.nih.gov/sra/), the accession number for our submissions are: PRJNA494774.

## Abbreviations

CDPK: Calcium-dependent protein kinase
DEGs: Differentially expressed genes
dpi: Post-inoculation
ET: Ethylene
ETI: Effector-triggered immunity
FLS2: LRR receptor-like serine/threonine-protein kinases
GCs: Giant cells
HSFs: Heat shock transcription factors
HSPs: Heat shock proteins
JA: Jasmonic acid
NADPH: Nicotinamide adenine dinucleotide phosphate
NBS-LRR: Nucleotide-binding site-leucine-rich repeat
NFS: Nematode feeding sites
PAMPs: Pathogen-associated molecular patterns
PCD: Programmed cell death
PTI: Pathogen-triggered immunity
RBOHs: Respiratory burst oxidases
RKNs: Root-knot nematodes
ROS: Reactive oxygen species
SA: Salicylic acid
SCN: Soybean cyst nematode
