# Supplementary figures and images for: Transcriptomic profiling of Solanum peruvianum LA3858 revealed a Mi-3-mediated hypersensitive response to Meloidogyne incognita
Source: BMC Genomics. 2020 Mar 23;21:250. doi: 10.1186/s12864-020-6654-5 (PMC7092525; doi:10.1186/s12864-020-6654-5)

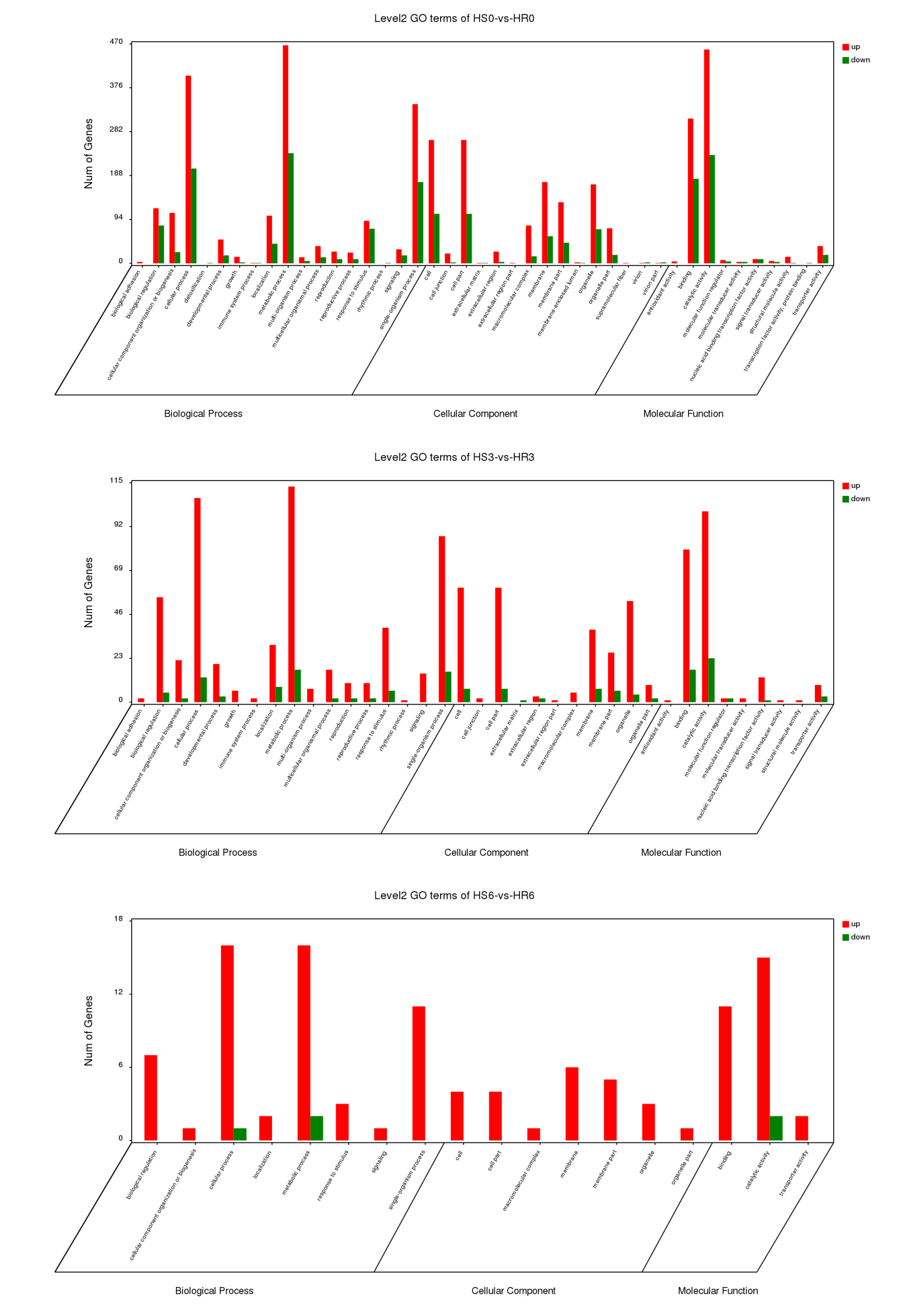

Supplement: Supplementary file 6 — Additional file 6: Figure S1 Level 2 GO terms identified for DEGs in 34 °C vs. 25 °C comparison at 0 (before inoculation), 3 and 6 dpi. [file 12864_2020_6654_MOESM6_ESM.png]

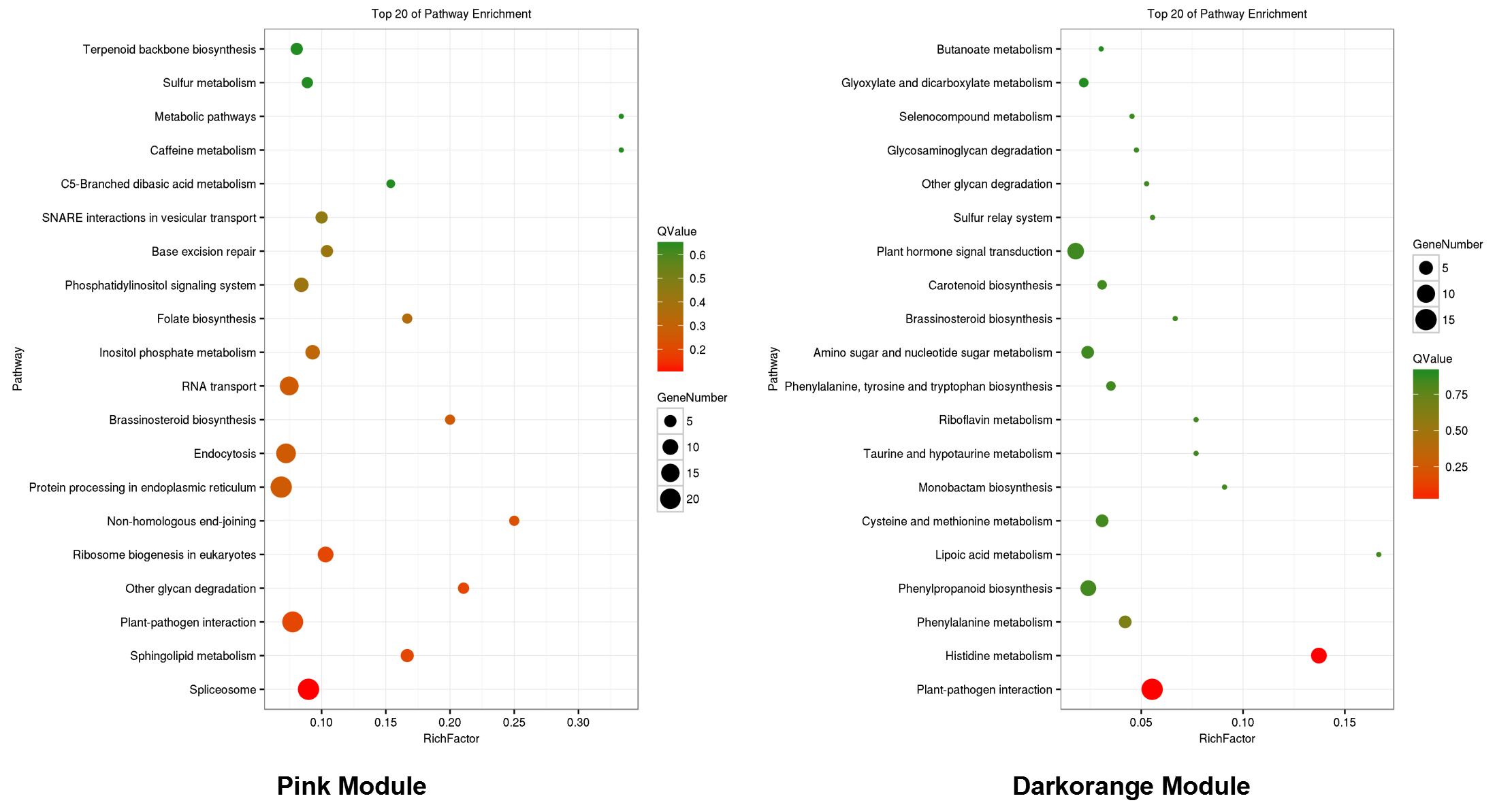

Supplement: Supplementary file 7 — Additional file 7: Figure S2 Top 20 significantly enriched pathways in the darkorange and pink modules according to WGCNA. [file 12864_2020_6654_MOESM7_ESM.png]

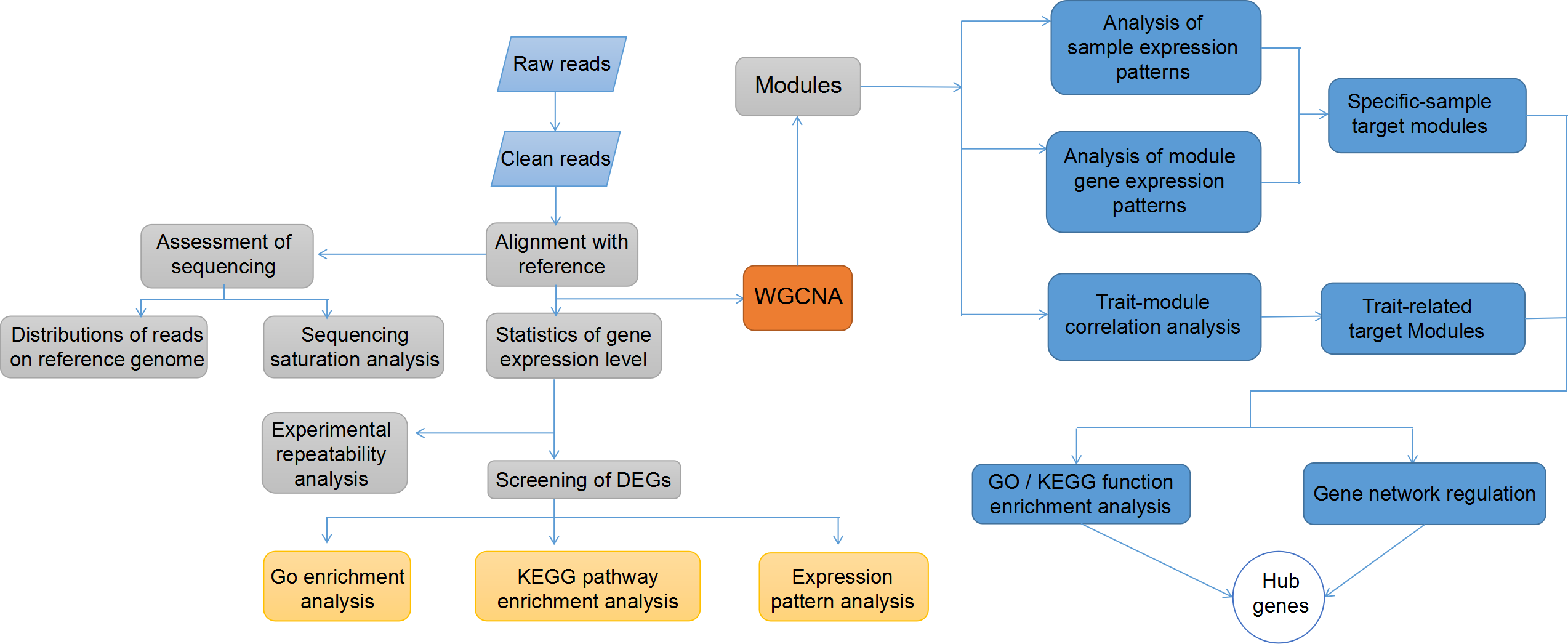

Supplement: Supplementary file 8 — Additional file 8: Figure S3 Description of all the analyses performed in this work. [file 12864_2020_6654_MOESM8_ESM.png]
